# Tribbles ortholog NIPI-3 and bZIP transcription factor CEBP-1 regulate a *Caenorhabditis elegans* intestinal immune surveillance pathway

**DOI:** 10.1186/s12915-016-0334-6

**Published:** 2016-12-07

**Authors:** Deborah L. McEwan, Rhonda L. Feinbaum, Nicholas Stroustrup, Wilhelm Haas, Annie L. Conery, Anthony Anselmo, Ruslan Sadreyev, Frederick M. Ausubel

**Affiliations:** 1 Department of Molecular Biology, Massachusetts General Hospital, Boston, MA USA; 2 Department of Genetics, Harvard Medical School, Boston, MA USA; 3 Department of Systems Biology, Harvard Medical School, Boston, MA USA; 4 Center for Cancer Research, Massachusetts General Hospital, Boston, MA USA; 5 Department of Pathology, Massachusetts General Hospital, Boston, MA USA; 6 Present Address: Center for Computational and Integrative Biology, Massachusetts General Hospital, Boston, MA USA

**Keywords:** Surveillance immunity, Tribbles-like kinase, C/EBP, *Pseudomonas aeruginosa*, Exotoxin A, Translational inhibition, *Caenorhabditis elegans*, Innate epithelial immunity, Lifespan machine

## Abstract

**Background:**

Many pathogens secrete toxins that target key host processes resulting in the activation of immune pathways. The secreted *Pseudomonas aeruginosa* toxin Exotoxin A (ToxA) disrupts intestinal protein synthesis, which triggers the induction of a subset of *P. aeruginosa*-response genes in the nematode *Caenorhabditis elegans*.

**Results:**

We show here that one ToxA-induced *C. elegans* gene, the Tribbles pseudokinase ortholog *nipi-3*, is essential for host survival following exposure to *P. aeruginosa* or ToxA. We find that NIPI-3 mediates the post-developmental expression of intestinal immune genes and proteins and primarily functions in parallel to known immune pathways, including p38 MAPK signaling. Through mutagenesis screening, we identify mutants of the bZIP C/EBP transcription factor *cebp-1* that suppress the hypersusceptibility defects of *nipi-3* mutants.

**Conclusions:**

NIPI-3 is a negative regulator of CEBP-1, which in turn negatively regulates protective immune mechanisms. This pathway represents a previously unknown innate immune signaling pathway in intestinal epithelial cells that is involved in the surveillance of cellular homeostasis. Because NIPI-3 and CEBP-1 are also essential for *C. elegans* development, NIPI-3 is analogous to other key innate immune signaling molecules such as the Toll receptors in *Drosophila* that have an independent role during development.

**Electronic supplementary material:**

The online version of this article (doi:10.1186/s12915-016-0334-6) contains supplementary material, which is available to authorized users.

## Background

A fundamental problem for all multicellular animals is that they must respond to invading pathogens while simultaneously tolerating or facilitating the growth of commensal microbes. Evidence has been mounting that metazoans can recognize pathogens by detecting the activity of so-called pathogen-encoded virulence effectors [1]. Although significant advances have been made in understanding how these immune triggers are sensed, it is poorly understood how they activate the defense responses that lead to the protection of host tissues from pathogen-inflicted damage and ultimately to the resolution of infection.

To investigate these issues, we study the nematode *Caenorhabditis elegans* which, when exposed to human pathogens, activates multiple discrete immune signaling pathways including an evolutionary conserved p38 MAPK pathway that is also critical for mammalian immunity [2]. For *C. elegans* infected with the gram-negative nosocomial pathogen *Pseudomonas aeruginosa*, deployment of these signaling pathways and resulting gene induction is directly correlated with bacterial virulence [3, 4], leading us to hypothesize that *P. aeruginosa* virulence factors may themselves trigger host immune gene expression. To test this theory, in previously published work, we screened for individual *P. aeruginosa* effectors that are capable of inducing a host immune response and discovered that exposure to Exotoxin A (ToxA) upregulates *C. elegans* immune genes [5]. ToxA is an extremely potent toxin of the AB class that inhibits protein translation by catalyzing the ADP-ribosylation of elongation factor 2, the same reaction catalyzed by diphtheria toxin from *Corynebacterium diphtheriae* and cholix toxin from *Vibrio cholerae* [6, 7]. The high level of toxicity of these enzymes has enabled their use as immunotoxins to treat a variety of cancers [8].

We determined that *C. elegans* recognizes ToxA independently of ToxA per se by detecting its enzymatic activity, translational inhibition [5]. Significantly, this immune activation is independent of physical microbial features called microbe- or pathogen-associated molecular patterns (MAMPs/PAMPs) or pattern recognition receptors, which are the traditionally studied mechanisms of pathogen recognition. Dunbar et al. [9] similarly discovered that inhibiting host translation stimulates the MAMP/PAMP-independent upregulation of the *C. elegans* ZIP-2 transcription factor resulting in *zip-2-*dependent gene induction. Mammalian cells are also able to sense pathogens by recognizing protein synthesis abnormalities; translational inhibitors secreted by *Legionella pneumophila* activate NF-κB and MAP kinase signaling and trigger the transcription of their target genes [10, 11], a subset of which are also upregulated at the protein level [12, 13]. Additional cellular processes commonly targeted by bacterial effectors are monitored through similar surveillance mechanisms [1, 14]. While a commonality of all these effector-triggered mechanisms is that they require either injury or modification to the host, the host genetic circuits that respond to these insults and act to protect against subsequent effector-mediated damage are only beginning to be understood. We therefore used the *C. elegans/*ToxA system to identify and characterize new components of surveillance signaling pathways.

Here, we investigate the genetic pathways that enable nematodes to mitigate the damaging effects of ToxA-mediated translational inhibition. Using an automated *C. elegans* Lifespan Machine [15], we show that the *C. elegans nipi-3* gene is required for animals to survive exposure to ToxA as well as to *P. aeruginosa*. NIPI-3 is a member of the highly conserved, functionally diverse Tribbles protein family, which, when mutated, has been linked to a variety of disorders related to cell signaling, immunity, metabolism, and cancer [16]. NIPI-3 has been previously shown to function upstream of p38 MAPK in the epidermis during fungal attacks [17] and, more recently, it has been determined to be required in multiple tissues during development (Kim et al., accompanying paper). Through partial loss-of-function and rescue studies, we find here that the *C. elegans* immune response against ToxA and *P. aeruginosa* is mediated in adult animals by intestinal NIPI-3 which, in contrast to epidermal NIPI-3, does not directly function in known *C. elegans* immune pathways but instead represses the activity of the bZIP C/EBP transcription factor CEBP-1*.*

## Results

### *nipi-3* mutants are hypersusceptible to translational inhibitors and *Pseudomonas aeruginosa*

We previously found that ToxA does not irreparably damage healthy *C. elegans* as wild type animals have the same longevity when feeding on an *E. coli* strain expressing *P. aeruginosa* PA14 ToxA as on control bacteria [5]*.* However, nematodes defective in immune signaling pathways, such as the p38 MAPK pathway, die rapidly when fed ToxA *E. coli* [5], implying that *C. elegans* normally resist this highly toxic enzyme through an effective host defense. We reasoned that genes upregulated in response to ToxA might be required to protect against or recover from toxin-induced damage and allow wild type worms to survive. Through genome-wide transcriptional profiling using Affymetrix GeneChips^©^, we previously identified 144 genes that were upregulated in wild type N2 *C. elegans* fed ToxA [5].

Using RNAi or mutant alleles corresponding to 125 of the most highly upregulated genes (Additional file 1: Table S1), we assayed for premature lethality in worms fed ToxA and found that the *nipi-3(fr4)* mutant exhibited the most significant reduction in lifespan (data not shown). In our previous microarray analysis, *nipi-3* was upregulated 4.3-fold in worms exposed to ToxA for 24 hours and was one of the two most highly induced kinases [5]. Whereas *nipi-3* null mutants arrest by larval development stage L3 (Kim et al., accompanying manuscript), *nipi-3(fr4)* is a fully viable hypomorphic allele, which, as described below, has allowed us to selectively address NIPI-3’s role in pathogen defense. *nipi-3(fr4)* contains a single mutation (I307N) in a conserved residue in the kinase domain [17].

**Fig. 1:**
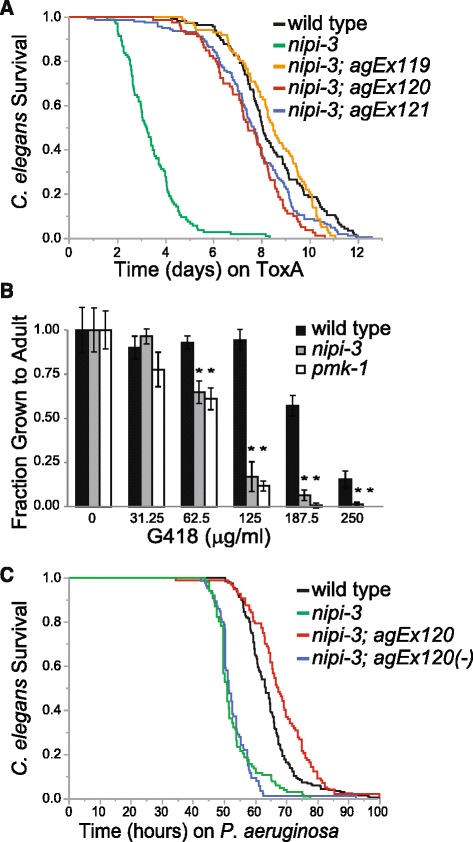
*nipi-3(fr4)* mutants have reduced resistance to ToxA, G418 and *Pseudomonas aeruginosa*. **a** Lifespans of wild type N2, *nipi-3(fr4)*, and three independent lines of *nipi-3(fr4)* expressing wild type *nipi-3p::nipi-3* fed *E. coli* expressing ToxA starting at the L4 stage. *P* < 0.0001 comparing *nipi-3(fr4)* and wild type (log-rank test). **b** Fraction of synchronized L1 worms that grew to at least young adult stage after 3 days at 20 °C on plates containing the indicated G418 concentration. Results shown are an average of four biological replicates. Error bars represent SD. *P < 0.05 compared to wild type animals at the given concentration (Student’s unpaired t*-*test). **c** Lifespans of wild type N2, *nipi-3(fr4)*, and *nipi-3(fr4)* expressing wild type *nipi-3p::nipi-3* fed on *P. aeruginosa* PA14. *nipi-3(fr4);agEx120(-)* indicates non-transgenic offspring of *nipi-3p::nipi-3* transgenic worms. *P* < 0.001 comparing *nipi-3(fr4)* and wild type (log-rank test). Number of animals scored for each condition was ≥ 80 (547 total; **a**) and > 85 (487 total; **c**). These are representative experiments of two independent experiments. Primary data for panel **b** are provided in Additional file 15

Using a *C. elegans* Lifespan Machine, a modified commercial flatbed scanner in conjunction with automated image analysis software [15], we analyzed the survival of the *nipi-3(fr4)* mutant feeding on ToxA and found that *nipi-3(fr4)* had a dramatically reduced lifespan on this food compared to wild type animals (Fig. 1a; *P* < 0.0001). Although *nipi-3(fr4)* has been reported to have a shortened lifespan [17], its longevity on ToxA was significantly shorter than on a control, non-pathogenic BL21 *E. coli* strain (Additional file 2: Figure S1a; *P* < 0.001), whereas the lifespans of wild type N2 worms on control BL21 *E. coli* or on ToxA were equivalent (Additional file 2: Figure S1a; *P* > 0.05) [5].

We previously showed that *C. elegans* immune-related signaling pathways, including the PMK-1 p38 MAPK pathway, are activated by ToxA-mediated translational inhibition rather than by ToxA itself. We therefore tested whether *nipi-3* mutants are also defective in their response to the protein synthesis inhibitor G418. Similar to *pmk-1* mutants, *nipi-3(fr4)* animals were more sensitive to G418 than wild type animals, indicating that *nipi-3* protects the host against translational inhibition and not an unrelated aspect of ToxA intoxication (Fig. 1b). Finally, we reasoned that *nipi-3* is likely to be required for *C. elegans* defense against *P. aeruginosa*, which produces ToxA. Indeed, *nipi-3(fr4)* mutants were hypersusceptible to *P. aeruginosa* in large-lawn automated assays in which *C. elegans* animals were unable to avoid being in contact with the bacterial lawn (Fig. 1c; *P* < 0.001). Reintroduction of wild type *nipi-3* expressed by its own promoter rescued the hypersusceptibility of *nipi-3(fr4)* to ToxA and *P. aeruginosa* (Fig. 1a, c).

To confirm that *nipi-3(fr4)* is not merely sensitive to any stressor, but is distinctly susceptible to translational inhibitors, we compared the ability of wild type and *nipi-3(fr4)* worms to recover from prolonged developmental arrest. Animals were arrested at the first larval stage and then starved for up to 14 days (L1 arrest or L1 diapause). Longer starvation reduces the number of animals that can recover upon feeding and grow to adults. Escaping this starvation-induced arrest requires the coordination of multiple pathways, including those required for general lifespan and stress responses [18]. In this assay, *nipi-3(fr4)* recovery was equivalent to wild type (Additional file 2: Figure S1b), indicating that *nipi-3(fr4)* is not broadly susceptible to any stressful condition.

### Immune genes are misregulated in *nipi-3(fr4)* mutants

We previously found that ToxA-sensitive mutants, such as *pmk-1(km25)*, misregulate pathogen-responsive genes [5], suggesting that *nipi-3(fr4)* animals will show similar transcriptional defects. However, unlike for *pmk-1*, complete loss of *nipi-3* function causes animals to arrest at the L2/L3 larval stage (Kim et al., accompanying paper) indicating that *nipi-3* is critical to both development and immunity. Thus, to understand the potential relationship between these processes and to dissect apart *nipi-3*’s apparent roles in both development and immunity, we performed genome-wide transcriptional profiling assays using Affymetrix GeneChips® comparing wild type, *nipi-3(fr4)*, and *pmk-1(km25)* animals that were fed control OP50 *E. coli.* Surprisingly, we found that a large number of genes were significantly upregulated in *nipi-3(fr4)* mutants: 282 genes were upregulated in *nipi-3(fr4)*, whereas, for comparison, only 8 genes were induced in *pmk-1(km25)* (Additional file 3: Table S2). Gene ontology (GO) term analysis showed that transcripts upregulated in *nipi-3* mutants were enriched for processes involved in immunity, similar to the GO terms enriched among the 43 genes downregulated in *pmk-1(km25)* animals (Additional file 3: Table S2), suggesting *nipi-3* may be a negative immune regulator. The only other overrepresented GO terms among the *nipi-3(fr4)*-upregulated genes involved flavonoid processes (Additional file 3: Table S2) due to the presence of 9 UDP-glucuronosyltransferases which, in addition to modifying flavonoids in plants, metabolize and detoxify xenobiotic compounds in metazoans [19, 20]. There were no enriched GO categories for the 71 genes downregulated in *nipi-3(fr4)* or the 8 genes upregulated in *pmk-1(km25)* animals. These gene expression results indicate that, similar to *pmk-1(km25)*, the *nipi-3(fr4)* allele is specifically defective in immune processes and that we can use *nipi-3(fr4)* to study the role of *nipi-3* in pathogen defense separately from its role in development.

To expand on the microarray results and test the effect of *nipi-3(fr4)* on immune gene expression under different conditions, we utilized a NanoString^©^ codeset containing 118 *C. elegans* genes involved in immune- and stress-related responses (Additional file 4: Table S3). NanoString analysis recapitulated the microarray gene expression changes observed between wild type and *nipi-3* mutant animals under normal growth conditions. We confirmed that 26 of the 27 codeset genes predicted to be *nipi-3*-dependent from the microarray data were similarly affected in the NanoString analysis; the remaining gene was upregulated 1.9× (false discovery rate (FDR) < 1 × 10^–2^), falling just below the two-fold cutoff. An additional 13 NanoString codeset genes that were not identified as being differentially regulated by microarray analysis were also statistically significantly up- or downregulated in *nipi-3(fr4)*, suggesting that the microarray analysis is either less sensitive or more stringent than NanoString.

We next asked whether immune- and stress-related genes are misregulated in *nipi-3(fr4)* mutants fed *P. aeruginosa* or ToxA, potentially explaining the hypersusceptibility of *nipi-3(fr4)* to these conditions. Of the 118 codeset genes, 49 were differentially expressed when comparing *nipi-3(fr4)* and wild type animals exposed to *P. aeruginosa* and 27 were affected in *nipi-3(fr4)* compared to wild type when fed ToxA (Additional file 4: Table S3)*.* In general, genes regulated by *nipi-3* were differentially expressed under multiple conditions (Additional files 4, 5: Table S3, Figure S2a); for example, 77% of the genes altered in *nipi-3(fr4)* on OP50 were also altered in *nipi-3(fr4)* on *P. aeruginosa*. We confirmed the microarray and NanoString gene expression changes for a subset of genes using qRT-PCR to analyze wild type and *nipi-3(fr4)* animals fed *P. aeruginosa*, ToxA, or their respective control bacteria (Additional file 6: Figure S3a, b).

In support of the hypothesis that *nipi-3* is an immune regulator, 44% (156) of the genes up or down regulated in *nipi-3(fr4)* animals on control OP50 bacteria were also responsive to ToxA or the translational inhibitor Hygromycin B (Additional file 5: Figure S2b; *P <* 1 × 10^–95^) as shown by comparing the current microarray dataset with our previous characterization of the wild type ToxA/Hygromycin B response [5]. In addition, genes differentially expressed in *nipi-3(fr4)* fed *P. aeruginosa* or ToxA compared to wild type animals on the same food were enriched for pathogen*-*response genes (Additional files 4, 5, 7: Tables S3, Figure S2c, and Table S4; *P* < 5 × 10^–6^). Taken together, these data demonstrate that *nipi-3* is necessary for the proper expression of immune genes in both unstressed and infected animals.

### ToxA induces defense proteins in wild type but not in *nipi-3(fr4)* animals

We next sought to determine the role of NIPI-3 on the host’s protein response to translational inhibitors since the extent to which mRNA up or downregulation affects protein expression under these conditions is unknown. We performed a large-scale quantitative proteomic analysis comparing wild type and *nipi-3(fr4)* animals fed on ToxA or control BL21 *E. coli*. This analysis detected a total of 7535 expressed proteins that map to 7444 unique *C. elegans* genes, representing approximately half of the genes expressed in adult animals [21].

**Fig. 2:**
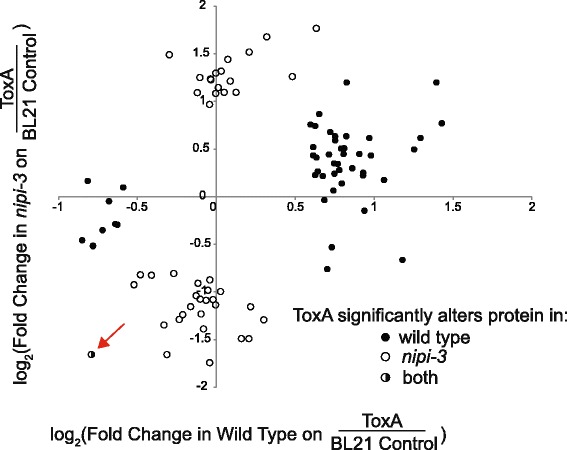
Different proteins are up and downregulated in wild type N2 and *nipi-3(fr4)* animals following ToxA exposure. Change of protein abundance in wild type N2 and *nipi-3(fr4)* animals following 24 hours feeding on *E. coli* expressing ToxA starting at the L4 stage as compared to animals fed control BL21 food. Only proteins significantly up or downregulated in wild type N2 and/or *nipi-3(fr4)* are included. The red arrow points to the only protein (T15B7.1) significantly altered in both N2 and *nipi-3(fr4)* animals. Results shown are an average of two biological replicates. Primary data are provided in Additional file 8: Table S5

We first characterized the protein changes that occur in wild type animals under translational stress. Of the 50 proteins that significantly changed in wild type upon ToxA exposure, 40 were upregulated and related to immunity based on GO term analysis (Additional file 8: Table S5). The induction of immune-related factors by ToxA was consistent with our previously published analysis of ToxA-mediated RNA changes [5]. However, unlike wild type animals, exposing *nipi-3(fr4)* mutants to ToxA caused more proteins to be downregulated than upregulated, 26 versus 17, respectively (Additional file 8: Table S5). There was almost no overlap between the *nipi-3(fr4)* and wild type responses to ToxA: only one protein was downregulated in both strains (red arrow, Fig. 2) and there were no common upregulated proteins. There were no enriched GO terms among the proteins up or downregulated by ToxA in *nipi-3(fr4)* consistent with the *nipi-3(fr4)* mutant mounting an uncoordinated, and ultimately ineffective, response to the toxin.

To directly address the relationship between transcript and protein changes in the presence of ToxA, we compared the proteomic data to the transcriptional profiling analysis that we previously performed using the same ToxA and control strains [5]. Of the 174 transcripts induced or repressed by ToxA in wild type worms, we identified 37 corresponding proteins and, of those, 15 (41%) were significantly affected by ToxA (Additional file 9: Figure S4a). Eight of the 10 RNAs with the highest ToxA induction were also elevated at the protein level. To understand how a protein inhibitor impacts the relationship between RNA and protein expression, we performed the same type of analysis using unstressed conditions lacking translational inhibitors. Specifically, we analyzed transcriptional and proteomic changes in *nipi-3(fr4)* compared to wild type animals fed on control *E. coli* (Additional file 10: Table S6) [5]. While 7 of the 10 RNAs with the highest induction in *nipi-3(fr4)* also had elevated protein levels, overall, there was less correlation between RNA and protein changes as only 12% (23/188) of *nipi-3(fr4)*-affected RNAs showed significantly altered protein expression. In addition, there were 39 proteins differentially expressed in *nipi-3(fr4)* that were unaffected at the RNA level (Additional file 9: Figure S4b). One interpretation for the poorer correlation in the *nipi-3*/wild type control analysis versus the ToxA/BL21 analysis is that RNAs induced during translational disruption have less post-transcriptional regulation than RNAs in unstressed conditions. However, a caveat of the *nipi-3* comparison is that different *E. coli* strains were used as controls in the transcriptomic and proteomic analyses (OP50 vs. BL21, respectively).

### Intestinal *nipi-3* defends against intestinal pathogens

**Fig. 3:**
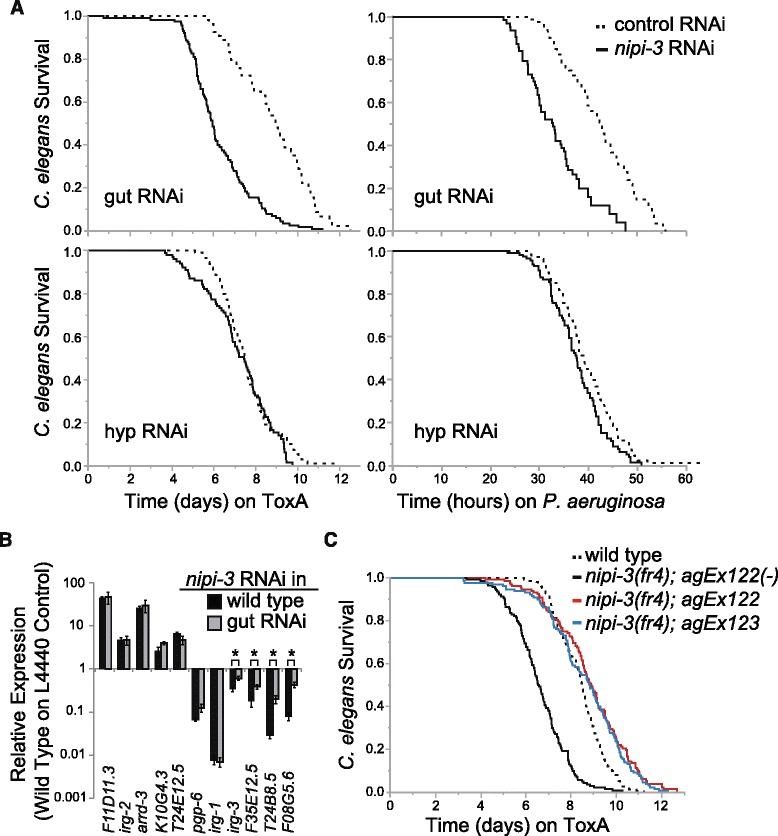
Intestinal *nipi-3* expression is necessary and sufficient for resistance against ToxA and *P. aeruginosa.* **a** Lifespans of gut or hypodermal RNAi strains fed *E. coli* expressing ToxA (left) or *P. aeruginosa* PA14 (right) following either *nipi-3* or L4440 vector control RNAi. *P* < 0.001 for gut RNAi assays; *P* > 0.02 for hyp RNAi assays (log-rank test). **b** qRT-PCR comparison of L4 animals following *nipi-3* RNAi started at the L1 stage. Results shown are an average of four biological replicates, each normalized to the corresponding wild type L4440 control values. Error bars represent SEM. *P < 0.05 compared to corresponding wild type animals (Student’s unpaired t-test). **c** Lifespans of wild type N2, two independent lines of *nipi-3(fr4)* expressing intestinal *vha-6p::nipi-3*, and the non-transgenic offspring of *nipi-3(fr4);agEx122* fed *E. coli* expressing ToxA. Number of animals scored for each condition was > 50 (373 total; **a** ToxA), ≥ 145 (656 total; **a** *P. aeruginosa*), and ≥ 130 (558 total; **c**). These are representative experiments of four (**a** ToxA), three (**a** *P. aeruginosa*), or two (**c**) independent experiments. Primary data for panel **b** are provided in Additional file 15

To confer resistance against the intestinal pathogen *P. aeruginosa*, *nipi-3* must act directly or indirectly in the intestine. NIPI-3 is expressed in multiple tissues in the adult *C. elegans* and was previously shown to function in the hypodermis but not the intestine to defend against the hypodermal fungal pathogen *D. coniospora* [17]. To determine whether the ToxA response is mediated by intestinally-expressed NIPI-3 or a systemic signal derived from hypodermally-expressed NIPI-3 and transported to the intestine, we knocked down *nipi-3* in either the intestine or hypodermis using tissue-specific RNAi *C. elegans* strains. We confirmed the tissue-specificity of these strains with bacterial RNAi clones targeting different tissues. We did not observe any silencing phenotypes in the gut-specific RNAi strain for RNAi clones targeting genes in the hypodermis, body wall muscle, or germline, but we did detect low levels of RNAi silencing in non-hypodermal tissues in the hypodermal-specific RNAi strain (Additional file 11: Table S7). Knocking down *nipi-3* in a gut-specific RNAi strain made animals hypersusceptible to ToxA and *P. aeruginosa* (Fig. 3a top; *P* < 0.001 for each assay), whereas knocking down *nipi-3* in the hypodermal RNAi strain did not result in increased ToxA susceptibility compared to control RNAi (Fig. 3a bottom; *P* > 0.02 for each assay), even though there was a low level of silencing in the intestine hypodermal RNAi strain (Additional file 11: Table S7). To confirm that intestinal *nipi-3* is responsible for the gene expression changes observed in Additional files 3, 4, and 6: Tables S2, S3, and Figure S3, we performed qRT-PCR of infection-related genes in either wild type or gut-specific RNAi animals following *nipi-3* RNAi. In both strains, five of the transcripts were upregulated and six were downregulated (Fig. 3b). Some transcripts showed a larger difference in wild type animals than in the gut-specific RNAi strain, which was potentially due to a difference in RNAi efficiency or indicates *nipi-3* has some activity in a non-gut tissue.

Finally, we tested whether NIPI-3 is also required in additional tissues by rescuing NIPI-3 only in the intestine of *nipi-3(fr4)* mutants. Expressing intestinal NIPI-3 was sufficient to rescue the *nipi-3(fr4)* ToxA defect (Fig. 3c). Taken together, the data in Fig. 3 show that intestinal NIPI-3 is both necessary and sufficient to mediate *C. elegans* ToxA defense mechanisms. Importantly, in contrast to knocking down *nipi-3* systemically, removing *nipi-3* only in the intestine did not result in a shortened lifespan on control food, and in fact appeared to enhance the lifespan (Additional file 2: Figure S1c). This latter experiment emphasizes that the role of *nipi-3* in pathogen defense can be separated from its lifespan and developmental effects.

### Intestinal *nipi-3* is not a primary component of known ToxA-response pathways including PMK-1 p38 or KGB-1 JNK-like MAP kinase

We next asked whether intestinal NIPI-3 is a component of known immune signaling pathways. Previous work has shown that, in the hypodermis, NIPI-3 functions upstream of the PMK-1 p38 MAPK pathway and between the protein kinase C TPA-1 and BiP/GRP78 chaperone HSP-3 [22, 23]. However, unlike *nipi-3(fr4)* animals, *tpa-1* and *hsp-3* mutants had a normal lifespan on ToxA (Additional file 12: Figure S5; *P* > 0.4 comparing ToxA and control *E. coli* for each strain) indicating that NIPI-3 has different functions in the hypodermis and intestine.

**Fig. 4:**
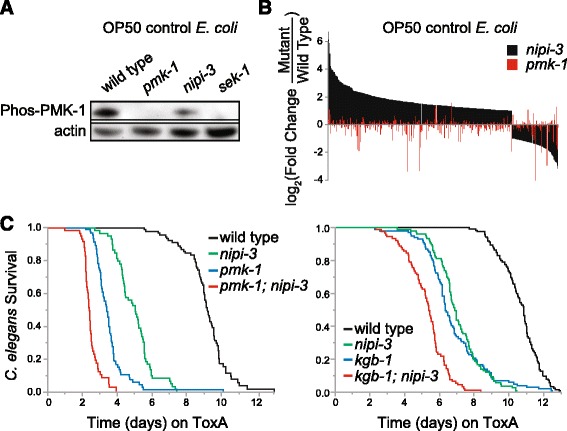
NIPI-3 is not immediately up or downstream of PMK-1 p38 MAPK or KGB-1 JNK-like pathways. **a** Western blot analysis of phosphorylated PMK-1 in L4 animals of the indicated genotype raised on control OP50 *E. coli*. **b** Transcript fold changes determined by microarray analysis of *nipi-3(fr4)* or *pmk-1(km25)* animals as compared to wild type N2 raised on OP50 *E. coli*. Only genes significantly altered in *nipi-3(fr4)* are shown. **c** Lifespans of wild type N2 and *pmk-1(km25)* (left) or *kgb-1(km21)* (right) fed *E. coli* expressing ToxA following either *nipi-3* or L4440 vector control RNAi. *P* < 0.001 comparing *pmk-1* and *pmk-1, nipi-3* and comparing *kgb-1* and *kgb-1; nipi-3* (log-rank test). Number of animals scored for each condition was ≥ 55 (278 total; **c** right) and > 90 (424 total; **c** left). These are representative experiments of two (**c** left) or three (**c** right) independent experiments. Primary data for panel **b** are provided in Additional file 3: Table S2

In contrast to *hsp-3* and *tpa-1*, *pmk-1* mutants die rapidly on ToxA [5] and the mammalian Tribbles homolog Trib2 is required for p38 phosphorylation in mammalian cells [24], suggesting that NIPI-3 and PMK-1 may function together. If NIPI-3 indeed functions solely upstream of or in conjunction with PMK-1, we reasoned that PMK-1 phosphorylation levels should be similar in *nipi-3(fr4)* animals to the levels observed in *sek-1* (MAPKK) mutants [25]. However, whereas PMK-1 phosphorylation is apparently decreased in *nipi-3(fr4)* compared to wild type worms, it is still present at a significantly higher level than in a *sek-1* mutant (Fig. 4a). We also tested whether *pmk-1* and *nipi-3* regulate common downstream genes as would be predicted if they are components of the same pathway. From our microarray analysis of animals on control OP50 *E. coli*, there was almost no overlap between *nipi-3*- and *pmk-1*-regulated genes (Fig. 4b); only 5 of the 101 genes downregulated in *nipi-3(fr4)* were repressed in *pmk-1(km25)* and, of the 282 genes upregulated in *nipi-3(fr4)*, 3 were upregulated and 8 were downregulated in *pmk-1(km25)* (Additional file 3: Table S2). We additionally analyzed the protein expression of *nipi-3(fr4)* and *pmk-1(km25)* mutants exposed to ToxA and detected no proteins in common between the 43 upregulated in *nipi-3(fr4)* and the 7 induced in *pmk-1(km25)* when comparing each to wild type worms on ToxA (Additional file 10: Table S6). Somewhat surprisingly, 16 proteins were repressed in both *nipi-3(fr4)* and *pmk-1(km25)* from a total of 106 *pmk-1(km25)*-downregulated and 25 *nipi-3(fr4)*-downregulated proteins.

Finally, consistent with NIPI-3 and PMK-1 mediating separate signaling pathways, we found that animals lacking both *nipi-3* and *pmk-1* had an increased susceptibility to ToxA as compared to loss of either protein alone (Fig. 4c left; *P* < 0.001 for *pmk-1* and *pmk-1;nipi-3*). A caveat of this experiment, however, is that the lifespan of the *pmk-1;nipi-3* animals on control food was also shortened (Additional file 13: Figure S6a). Taken together, while there may be some cross-talk between the NIPI-3 and PMK-1 pathways, our gene expression and pathogen-susceptibility data are not consistent with a model in which NIPI-3 functions directly up or downstream of PMK-1.

We next focused on the KGB-1 JNK-like MAPK pathway since, like *nipi-3*, *kgb-1* is required for resistance to *P. aeruginosa* and acts in parallel to *pmk-1* (Fig. 6) [25]. In addition, mammalian Trib2 has a role in JNK phosphorylation [24]. While we found that *kgb-1* mutants were hypersusceptible to ToxA, loss of both *kgb-1* and *nipi-3* resulted in an additive ToxA effect compared to loss of either gene alone (Fig. 4c right; *P* < 0.001 comparing *kgb-1;nipi-3* and *kgb-1* or *nipi-3*). However, as with the *pmk-1* analysis, the lifespan for *kgb-1;nipi-3* on control *E. coli* was attenuated compared to loss of only *kgb-1* or *nipi-3* (Additional file 13: Figure S6b). Therefore, we asked whether *kgb-1(km21)* and *nipi-3(fr4)* regulate the same set of downstream genes. We determined that none of the eight ToxA-response genes assayed by qRT-PCR were regulated by both *kgb-1* and *nipi-3* on control *E. coli* and ToxA (Additional file 6: Figure S3b), consistent with KGB-1 and NIPI-3 acting in parallel.

Finally, the bzip transcription factor ZIP-2 and G-protein coupled receptor FSHR-1 regulate different subsets of ToxA-response genes and function separately from the PMK-1 and KGB-1 pathways (Fig. 6) [5]. As was the case with *pmk-1* and *kgb-1*, there was no overlap between the transcripts significantly altered in *zip-2* or *fshr-1* and *nipi-3* mutants on control *E. coli* and *P. aeruginosa* (Additional file 6: Figure S3a). In addition, *zip-2* and *fshr-1* mutants were much less sensitive to ToxA than *nipi-3(fr4)* (Additional file 6: Figure S3c; data not shown for *fshr-1*), making it unlikely that either ZIP-2 or FSHR-1 are in a linear pathway with NIPI-3.

### *nipi-3* genetically interacts with the C/EBP bZIP transcription factor *cebp-1* to promote ToxA resistance

**Fig. 5:**
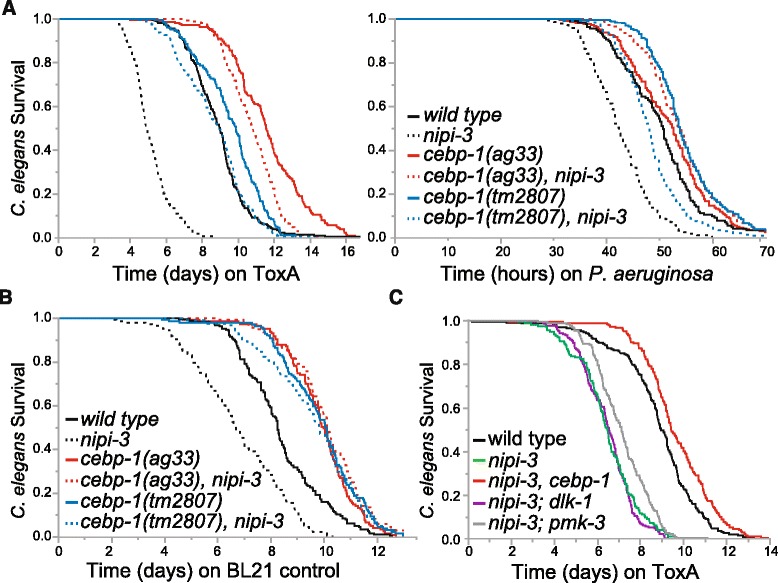
*nipi-3(fr4)* survival defects are suppressed by loss of *cebp-1* but not loss of *pmk-3* or *dlk-1*. Lifespans of the indicated strains on *E. coli* expressing ToxA (**a** left, **c**), *P. aeruginosa* PA14 (**a** right), or control BL21 *E. coli* (**b**). *nipi-3(fr4)* was used for all assays. *cebp-1*, *dlk-1*, and *pmk-3* were inhibited by RNAi in **c**. Number of animals scored for each condition was > 160 (1178 total; **a** right), > 140 (1239 total; **a** left), > 120 (875 total; **b**), and > 160 (937 total; **c**). These are representative experiments of two independent experiments

Based on the epistasis analyses, NIPI-3 represents either the first identified factor of a new signaling pathway acting in parallel to PMK-1 or it functions to modulate multiple pathways and coordinate the downstream effects of different initial inputs. If NIPI-3 represents a separate branch of immune signaling, we reasoned that we should be able to identify distinct factors functioning downstream of *nipi-3*. To test this, we mutagenized *nipi-3(fr4)* animals with EMS and identified 22 mutants with increased resistance to ToxA (see Methods for details). We selected two mutants with the strongest phenotypes from two independent pools for further analysis. Through whole genome sequencing, we found that both mutants contained an A246V mutation in the DNA binding domain of the C/EBP bZIP transcription factor CEBP-1. When exposed to ToxA or *P. aeruginosa*, both the newly-discovered *cebp-1(ag33)* allele as well as a previously-isolated *cebp-1* deletion allele (*tm2807*), completely suppressed the *nipi-3(fr4)* defect in both infection conditions as compared to wild type animals (Fig. 5a). However, the single *cebp-1* mutants survived longer on ToxA than wild type worms and the double *cebp-1, nipi-3* mutants were slightly shorter lived on ToxA than their single *cebp-1* mutant counterparts (Fig. 5a left; *P* < 0.001) suggesting that *nipi-3* may have a minor additional function in a non-*cebp-1* pathway to protect against ToxA. *cebp-1* mutants also suppress the *nipi-3(fr4)* lifespan defect as the lifespans of the single *cebp-1* and double *cebp-1, nipi-3* mutants were equivalent on control *E. coli* (Fig. 5b; *P* > 0.05).

CEBP-1 is expressed in pharyngeal, neuronal and intestinal cells [26], and is known to function in neurons during axon regeneration [27]. Therefore, we asked whether *cebp-1* acts in the intestine to mediate ToxA defenses. To test this, we used the gut-specific RNAi strain described above and compared its susceptibility to ToxA following knockdown of either *nipi-3* or *cebp-1* or of both genes simultaneously. While loss of intestinal *nipi-3* resulted in ToxA hypersusceptibility, animals with co-knockdown of intestinal *nipi-3* and *cebp-1* had an equivalent lifespan on ToxA as those lacking only intestinal *cebp-1 (*Additional file 14: Figure S7a; for *nipi-3, cebp-1* vs. *cebp-1* RNAi, *P* = 0.95 by log-rank test and 0.4 by Wilcoxon test; for *nipi-3* vs. *cebp-1* RNAi, *P* = 0.089 by long-rank test and 0.0004 by Wilcoxon test), indicating that CEBP-1 mediates the ToxA defense by functioning in the intestine.

In neurons, CEBP-1 acts downstream of the DLK-1/PMK-3 p38 MAPK pathway [27] and so we determined whether *dlk-1* and *pmk-3* are also involved in the NIPI-3 intestinal immune pathway. We found that the PMK-3 pathway is not necessary for the ToxA defense since, unlike RNAi-mediated knockdown of *cebp-1*, RNAi of *dlk-1* or *pmk-3* did not suppress the ToxA sensitivity of *nipi-3(fr4)* (Fig. 5c).

Finally, since loss of *nipi-3* results in immune gene misexpression (Additional files 3, 4, 6: Tables S2, S3 and Figures S3), we tested whether *cebp-1* rescues *nipi-3*’s pathogen survival phenotype by restoring wild type gene expression levels during ToxA intoxication. Using qRT-PCR, we compared wild type animals with single *nipi-3* and *cebp-1* mutants as well as double *nipi-3, cebp-1* mutants. For some genes, such as *T24B8.5*, the *nipi-3(fr4)* defect was partially rescued in the double *nipi-3, cebp-1* mutants but, for others, such as *F11D11.3*, the double mutants showed stronger misregulation than *nipi-3(fr4)* alone (Additional file 14: Figure S7b). A major limitation of this experiment, however, is that it is unknown which *nipi-3*-dependent genes are essential for the ToxA response. We attempted to identify critical effector proteins acting downstream of NIPI-3/CEBP-1 by asking whether any of the individual proteins differentially regulated in *nipi-3(fr4)* could account for the pathogen sensitivity of these mutants. To mimic the defects in *nipi-3(fr4)*, for proteins downregulated in *nipi-3(fr4)*, we knocked down the corresponding genes by RNAi in wild type worms and assayed for worms that phenocopied the *nipi-3(fr4)* ToxA susceptibility. We similarly inhibited the proteins that were upregulated in *nipi-3(fr4)* using RNAi in *nipi-3(fr4)* mutants and examined whether these animals survived longer on ToxA. However, we found no major changes in ToxA sensitivity following these knockdowns (data not shown).

## Discussion

In this study, we find that NIPI-3 is an essential component of the *C. elegans* defense against the translational inhibitor ToxA as well as *P. aeruginosa*, the bacterial pathogen that secretes ToxA. Unlike other previously described immune signaling genes, including *pmk-1*, *nipi-3* is upregulated by ToxA. Perhaps counterintuitively, loss of *nipi-3* results in the upregulation of many ToxA-inducible genes in the absence of translational inhibition. This does not occur for the ToxA-sensitive *pmk-1* mutant in which immune and stress genes are overall downregulated compared to wild type animals and suggests that NIPI-3 may have a specialized inhibitory role in responding to translational inhibitors.

NIPI-3 plays critical roles in both development and immunity, analogous to other key innate immune signaling molecules such as the Toll receptors in *Drosophila* [28] and *bar-1* β-catenin in *C. elegans* [29]. While null mutations in *nipi-3* cause animals to arrest by the L3 stage (Kim et al., accompanying paper), the use of the fully viable *nipi-3(fr4)* hypomorphic allele has allowed us to specifically investigate NIPI-3’s role in pathogen defense. Through tissue-specific RNAi and rescue experiments, we determined that NIPI-3-mediated ToxA and *P. aeruginosa* defenses are controlled in adult animals by intestinally expressed *nipi-3*, consistent with *P. aeruginosa* being a *C. elegans* intestinal pathogen*.* Previous reports show that hypodermal but not intestinal expression of *nipi-3* is important for survival against the hypodermal pathogen *D. coniospora* [17]. A simple explanation of these data is that, in late larval stage and adult animals, following the requirement for NIPI-3 in development, NIPI-3 is only necessary in the tissue that is under pathogenic attack, which implies that it functions autonomously to mediate local immune responses. Alternatively, it is also possible that intestinally- and hypodermally-expressed *nipi-3* regulate different immune mechanisms, with the intestinal responses effective against ToxA-like molecules and the hypodermal responses effective against distinct pathogenic mechanisms of *D. coniospora.* Supporting the hypothesis that *nipi-3* may have unique functions in different tissues, we show here that the immune signaling molecules *hsp-3* and *tpa-1*, which function in the hypodermal NIPI-3 pathway [22, 23], are dispensable for mounting ToxA resistance in the intestine, even though, as with *nipi-3*, they are expressed in the intestine [30, 31].

In contrast to *hsp-3* and *tpa-1*, *pmk-1* mutants are hypersensitive to ToxA and the translational inhibitor G418 but *nipi-3* does not appear to function strictly upstream or downstream of p38 MAPK in response to ToxA. Microarray analysis of *pmk-1* and *nipi-3(fr4)* L4 animals, the stage exposed to ToxA in our standard assay, revealed that the gene expression signature in these two mutants is remarkably different on control OP50 bacteria. However, there may be some interplay between *nipi-3* and *pmk-1* because the overall level of activated PMK-1 was reduced in L4 *nipi-3(fr4)* animals. Upon exposure to ToxA, most of the proteins that changed in the young adult *nipi-3(fr4)* mutant animals exhibited an increase over wild type, whereas the opposite was true for the *pmk-1* mutant animals, where many more proteins were downregulated. While the majority of overall proteins differentially expressed in *nipi-3(fr4)* or *pmk-1(km25)* on ToxA did not overlap, a subset of proteins downregulated in *nipi-3(fr4)* mutant animals were also reduced in the *pmk-1* mutant, suggesting that either these are a signature of worms dying from ToxA exposure or there is a commonality in the *nipi-3* and *pmk-1* responses to ToxA. However, we argue that *nipi-3* and *pmk-1* primarily function in parallel for their response to the toxin because reduction of *nipi-3* activity by RNAi in the *pmk-1* null mutant resulted in increased hypersusceptibility to ToxA. Interestingly, and in contrast to our *P. aeruginosa* data, *nipi-3* acts upstream of the p38 MAPK signaling cassette (*tir-1/nsy-1/sek-1/pmk-1*) in the hypodermis to regulate the induction of anti-microbial peptides in response to *D. coniospora* [17, 22, 32]. In addition, mutations in the p38 PMK-1 MAPK pathway suppress *nipi-3*-dependent developmental arrest and *nipi-3* null mutant animals hyperactive PMK-1 at early larval stages (Kim et al., accompanying paper). Combining these studies, it is clear that there are multiple regulatory interactions between *nipi-3* and *pmk-1* across different developmental stages and tissues and these likely contribute to the varied functions of NIPI-3.

**Fig. 6:**
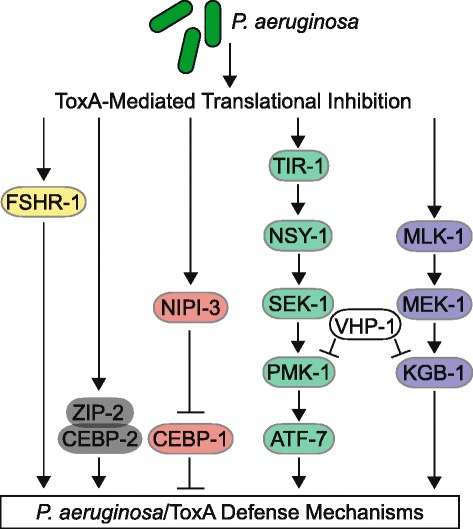
Model of major *C. elegans* pathways involved in ToxA defenses

For adult *nipi-3(fr4)* animals exposed to *P. aeruginosa* or ToxA, their shortened lifespan can be completely suppressed by loss of the C/EBP bZIP transcription factor *cebp-1*. Removal of *cebp-1* does not cause *C. elegans* to be hypersusceptible to ToxA, suggesting it normally functions as a negative regulator. Therefore, the explanation for *nipi-3(fr4)* lethality on ToxA is that CEBP-1 function is now increased, making NIPI-3 a negative regulator of CEBP-1 (Fig. 6)*.* In support of this model, Kim et al. (accompanying paper) found that *cebp-1* transcription is increased in the *nipi-3* mutant and there is a remarkable overlap in the gene classes enriched in *cebp-1* targets and those repressed by *nipi-3*, specifically in the “stress cluster” (Kim et al., accompanying paper)*.* Finally, other Tribbles proteins are also known to promote degradation of target proteins, including MAPKs and C/EBP proteins [16]. It should be noted that cebp-1 mRNA and protein were upregulated in *nipi-3(fr4)* animals, although these inductions were not statistically significant.

While the *cebp-1* ag33 allele results in a relatively minor change in CEBP-1, this mutation has been isolated independently at least three times: twice in this study and once by Bounoutas et al. [33] in a neuronal mutagenesis screen that identified *cebp-1(u819)* as a suppressor of the microtubule disrupter colchicine, which normally causes a general decrease in protein expression in *C. elegans* touch receptor neurons. Unlike in our study, mutations in the PMK-3 p38 MAPK phenocopy the loss of neuronal *cebp-1*. For both our study and that of Bournoutas et al. [33], regardless of the upstream pathway, CEBP-1 is detrimental to mitigating the effects of translational inhibitors, either ToxA or colchicine. Interestingly, another C/EBP transcription factor, *cebp-2*, has also recently been shown to play an important role in *C. elegans* translational defense. However, unlike CEBP-1, CEBP-2 promotes ToxA and *P. aeruginosa* protective mechanisms by acting as a heterodimeric transcription factor with ZIP-2 [34]. The ability of CEBP proteins to both promote and repress protective host defenses against the same pathogen demonstrates the range of CEBP immune functions and raises the question of whether, for example, CEBP-1 could be a positive effector under a different condition.

The mechanism by which translational inhibitors activate the NIPI-3/CEBP-1 pathway remains an open question. Dunbar et al. [9] discovered that ZIP-2 accumulation during a *P. aeruginosa* infection is mediated by open reading frames in the *zip-2* 5’UTR (uORFs). This pathogen-mediated increase in translation is potentially similar to the response of yeast GCN4 and mammalian ATF4 to nutritional stresses and translational inhibition [35]. However, we did not observe uORFs in the *nipi-3* 5’UTR. Moreover, proteins induced by ToxA in wild type worms were not enriched for uORFs, implying that additional activation mechanisms must be present. One such strategy may be linked to transcript abundance; Ivanov and Roy [13] found that the most abundant transcripts could bypass the general protein synthesis block caused by *L. pneumophila* translational inhibitors. However, a similar study by Asrat et al. [12] found that translation of the IL-1β cytokine was independent of its transcript stability, suggesting that increasing a transcript’s concentration is not sufficient to ensure its protein expression. Another mechanism of post-transcriptional regulation could be mediated by a second protein such as a labile repressor that is not synthesized when translation is disrupted. This is observed at the transcriptional level in mammalian macrophages when *L. pneumophila* translational inhibitors block the production of new NFκB repressor IκB, resulting in increased gene expression [10]. It is possible that, if it exists, such a suppressor could be identified in further *C. elegans* screening.

Future studies are also likely to identify additional mechanisms for activating surveillance immune pathways and may identify strategies employed by the pathogens to suppress them. A striking finding from this study is the approximately 40% correlation between transcript and protein changes in the worms following ToxA exposure, which is comparable to other published studies analyzing a variety of conditions [36] and suggests that, after a 24 hour exposure, ToxA does not globally affect post-transcriptional processing. However, ToxA expressed by the *P. aeruginosa* strain PA01 is able to broadly reduce *C. elegans* protein synthesis [37], which may indicate that *P. aeruginosa* expresses effectors to inhibit the nematode’s ToxA defense.

## Conclusions

We have shown here that *nipi-3* enables hosts to survive pathogen-mediated damage to protein translation. NIPI-3 mediates post-developmental immunity in the intestine by acting as a negative regulator of the C/EBP transcription factor CEBP-1, which in turn negatively regulates protective immune processes. This pathway represents a new branch of intestinal innate immune signaling.

## Methods

### Strains

*C. elegans* were maintained using standard methods. *C. elegans* strains used in this study were: N2 (wild type), IG544 *nipi-3(fr4)*, KU25 *pmk-1(km25)*, MJ563 *tpa-1(k530)*, RB1104 *hsp-3(ok1083)*, AU0067 *kgb-1(km21)*, RB911 *fshr-1(ok778)*, ERT61 *zip-2(tm4248)*, KU4 *sek-1(km4)*, MGH167 *sid-1(qt9);alxIs6[vha-6p::SID-1::SL2::GFP]*, JM43 *rde-1(ne219);Is[wrt-2p::RDE-1]*, CZ8920 *cebp-1(tm2807)*, KU12 *dlk-1(km12)*, BS33830 *pmk-3(ok169)*, and CZ8920 *cebp-1(tm2807)*. The *C. elegans* strains created for this study were: AU0329 *nipi-3(fr4); agEx119(myo-2p::MCherry, nipi-3p::NIPI-3)*, AU0330 *nipi-3(fr4);agEx120(myo-2p::MCherry, nipi-3p::NIPI-3)*, AU0331 *nipi-3(fr4);agEx121(myo-2p::MCherry, nipi-3p::NIPI-3)*, AU0332 *nipi-3(fr4);agEx122(myo-2p::MCherry, vha-6p::NIPI-3)*, AU0333 *nipi-3(fr4);agEx123(myo-2p::MCherry, vha-6p::NIPI-3)*, AU0350 *nipi-3(fr4) cebp-1(ag33)*, AU0352 *cebp-1(ag33)*, and AU0351 *nipi-3(fr4) cebp-1(tm2807)*.

*nipi-3p::NIPI-3* was amplified from the fosmid UBC_f80B0723Q (source bioscience UK limited) with the primers 5’-TGTTACCTGAAAGTTGCGGA and 5’-CCCGATTCAACTGTTTCAGG. For *vha-6p::NIPI-3*, *NIPI-3* was amplified from the *nipi-3p::NIPI-3* fusion using the primers 5’- CTAAACTAGTGGGTATGGCTCGTACAAAATGC and 5’-CCCGATTCAACTGTTTCAGG and *vha-6p* was amplified from N2 genomic DNA with the primers 5’-GATATTGCCAGCATGCTCAACG and 5’-TCTAGATATGGGTTTTGGTAGGTTTTAGTCG. The *NIPI-3* and *vha-6p* fragments were ligated together in the presence of SpeI, XbaI, and T4 DNA ligase (NEB) and the expected sized fragment was gel purified with the QIAquick Gel Extraction kit (Qiagen). Constructs were injected into worms with the co-injection marker *myo-2p::MCherry*, and 1 KB ladder (NEB).

ToxA and BL21 *E. coli* control (an empty pet100 vector control) have been previously described [5]. All *P. aeruginosa* assays used the clinical isolate PA14 [38]. RNAi clones were obtained from the Ahringer RNAi library [39] and confirmed by sequencing.

### *C. elegans* lifespan/killing assays

*nipi-3* RNAi experiments were started from the L1 stage and mimicked the survival phenotype of *nipi-3(fr4)* mutants. All lifespan and killing assays were performed using the *C. elegans* Lifespan Machine [15] using worms and bacteria prepared as described [5], with the modification that *E. coli* plates were incubated for 24 hours at room temperature before use. All *P. aeruginosa* experiments utilized full lawn assays. The automated, high-resolution *P. aeruginosa* strain PA14 killing assays required significant modification of the image analysis software described in [15] to account for the disappearance, or ghosting, of dead animals, the relative opaqueness of the PA14 lawn, and a different plate type.

### Multiplexed quantitative proteomics

Approximately 10,000 L4 worms were plated per condition and collected after 24 hours at 25 °C. Worms were washed with M9, incubated for 15 minutes at room temperature to remove intestinal bacteria, washed again and flash frozen in liquid nitrogen. Worms were ground using a mortar and pestle on dry ice and resuspended in 50 mM HEPES pH 8.5, 3% SDS, and EDTA-free complete protease-inhibitor (Roche). Samples were spun down to collect the supernatant. Multiplexed quantitative proteomics was performed using TMT reagents and an Orbitrap Fusion mass spectrometer applying Simultaneous Precursor Selection-MS3 supported quantification [40, 41], see below. Differentially regulated proteins were determined by QSPEC (http://www.nesvilab.org/qspec.php/; version 1.2.2) [42] using a FDR < 0.05 and log_2_(Fold Change) > 0.585 or < –0.585.

For the mass spectrometer analysis, cells were lysed in a buffer containing 75 mM NaCl, 50 mM HEPES (pH 8.5), 10 mM sodium pyrophosphate, 10 mM NaF, 10 mM β-glycerophosphate, 10 mM sodium orthovanadate, 10 mM phenylmethanesulfonylfluoride, Roche Complete Protease Inhibitor EDTA-free tablets, and 3% sodium dodecyl sulfate. Lysis was achieved by passing cells 10 times through a 21-gauge needle. Lysates were further processed through reduction and thiol alkylation was followed by purifying the proteins using MeOH/CHCl_3_ precipitation. Protein digestion was performed with Lys-C and trypsin. Peptides were labeled with TMT-10plex reagents (Thermo Scientific) [43] and fractionated by basic pH reversed phase chromatography as described elsewhere [44]. Multiplexed quantitative proteomics was performed on an Orbitrap Fusion mass spectrometer (Thermo Scientifc) using a Simultaneous Precursor Selection-based MS3 method [41]. MS2 spectra were assigned using a SEQUEST-based [45] proteomics analysis platform [46]. Based on the target-decoy database search strategy [47] and employing linear discriminant analysis and posterior error histogram sorting, peptide and protein assignments were filtered to FDR of < 1% [46]. Peptides with sequences that were contained in more than one protein sequence from the UniProt database were assigned to the protein with most matching peptides [46]. TMT reporter ion intensities were extracted as that of the most intense ion within a 0.03 Th window around the predicted reporter ion intensities in the collected MS3 spectra. Only MS3 with an average signal-to-noise value of larger than 40 per reporter ion as well as with an isolation specificity [40] of larger than 0.75 were considered for quantification. A two-step normalization of the protein TMT-intensities was performed by first normalizing the protein intensities over all acquired TMT channels for each protein based to the median average protein intensity calculated for all proteins. To correct for slight mixing errors of the peptide mixture from each sample, a median of the normalized intensities was calculated from all protein intensities in each TMT channel and the protein intensities were normalized to the median value of these median intensities.

### Gene expression analyses

Microarray samples were prepared as previously described [5]. RMA normalized gene expression values were calculated using the R package ‘affy’ (Release 3.3) [48], while differential expression analysis was performed with the package ‘limma’ [49]. NanoString probe hybridization and data acquisition were performed according to manufacturer’s protocols and differentially regulated transcripts were determined by edgeR [50]. For the microarray and NanoString analyses, genes were considered differentially regulated at FDR < 0.05 and log_2_(Fold Change) > 1 or < –1. Quantitative RT-PCR was performed and analyzed as described [5] and *P* values were determined with an unpaired, two-tailed Student t-test.

### GO term analysis

Data was analyzed through the Gene Ontology Consortium (www.geneontology.org) [51] [52]. Terms were considered enriched if *P* < 1 × 10^–3^ and Fold Enrichment > 5.

### Immunoblot analysis

L4 worms raised on OP50 were washed with M9 to remove bacteria and resuspended in Laemmli sample buffer (BioRad). Samples were boiled, spun down, and supernatants were flash frozen in liquid nitrogen. Proteins were resolved on a 4–12% Bis-Tris SDS gel, transferred to a nitrocellulose membrane, and probed with anti-phospho-PMK-1 (Promega) or anti-actin (Abcam) antibodies.

### Larval starvation assay

L1 stage animals were synchronized by hypochlorite treatment and overnight hatching in M9 at room temperature. At the indicated day, approximately 100 animals were transferred to NGM plates seeded with OP50 bacteria and counted. Plates were incubated at room temperature for 2 or 3 days and scored for growth.

### Mutagenesis

Ethyl methane sulfonate mutagenesis of *nipi-3(fr4)* animals was carried out using standard methodology [53]. Fortuitously, we found that feeding *nipi-3(fr4)* on HT115 *E. coli* expressing *vhp-1* RNAi led to early stage larval arrest, animals were of undetermined developmental age but not larger than wild type L2 animals, and the arrested animals subsequently died within several days. This is in contrast to N2 wild type animals fed the same *vhp-1* RNAi expressing strain that showed delayed development compared to the control HT115 L4440 strain but matured to gravid adults within 2–3 days at 25 °C. F2 generation eggs from approximately 25,000 mutagenized *nipi-3(fr4)* haploid genomes were isolated by hypochlorite treatment and dropped directly onto *vhp-1* RNAi food. Animals were incubated at 25 °C for 2 days and L4, young adult or gravid adult animals were picked from the *vhp-1* RNAi to standard OP50 *E. coli* food. The recovered putative *nipi-3* suppressors were re-screened for sensitivity to ToxA in order to eliminate (1) RNAi defective mutants and (2) known suppressors of *vhp-1*, such as *mlk-1*, *mek-1*, *kgb-1* [54], and *pmk-3* [55], which have been previously shown to suppress the larval arrest of *vhp-1* mutants but do not suppress the ToxA sensitivity of *nipi-3*. For this ToxA secondary test, between 20 and 45 L4 progeny from each putative *nipi-3* suppressor were picked to ToxA and their survival at 25 °C was followed until all the un-mutagenized *nipi-3(fr4)* animals were dead, at which point 100% of N2 wild type animals were still alive. Two suppressors from independent pools of mutagenized animals were identified that restored *nipi-3(fr4)* mutant animals to wild type levels of ToxA resistance. Each of the two suppressors was back crossed to *nipi-3(fr4)* and at least 50 F2 recombinants which grew to maturity on *vhp-1* RNAi and exhibited wild type levels of ToxA resistance were pooled for DNA isolation. The corresponding genetic lesions were identified by next generation sequencing technology as previously described [56]. Methods were modified for sequencing on Illumina MiSeq according to the manufacturer’s instructions. The *nipi-3(fr4)* strain was also sequenced and used as the reference strain for identification of homozygous variants. During the process of backcrossing, the *nipi-3(fr4*) suppressors were both shown to be X-linked, which aided in the identification of relevant sequence variants.

## Additional files

Additional file 1: Table S1. Testing whether ToxA-responsive genes defend against ToxA. This table lists the genes that were previously found to be significantly induced by *E. coli* expressing ToxA [5] and were inhibited either by RNAi or with mutant alleles to determine whether they are necessary for ToxA resistance. (XLSX 15 kb)
Additional file 2: Figure S1. The ToxA defect of *nipi-3(fr4)* is not a result of general strain health. a. Lifespans of *nipi-3(fr4)* and wild type N2 fed *E. coli* expressing ToxA or the BL21 control. *P* < 0.001 comparing *nipi-3(fr4)* ToxA and BL21; *P* > 0.05 comparing N2 ToxA and BL21 (log-rank test). b. Fraction of *nipi-3(fr4)* or wild type N2 animals that grow within 2 or 3 days of being fed OP50 following starvation at room temperature for the indicated time. Results shown are an average of six biological replicates. Error bars represent SD. c. Lifespans of wild type N2 and MGH167 (gut RNAi) animals fed on BL21 control *E. coli* following either *nipi-3* or L4440 vector control RNAi. Number of animals scored for each condition was > 65 (286 total; a) and > 65 (332 total; c). These are representative experiments of four (a), three (c), or two (b) independent experiments. (PDF 435 kb)
Additional file 3: Table S2. Affymetrix microarray analysis of wild type N2, *nipi-3(fr4)*, and *pmk-1(km25)* gene expression. This table lists the genes differentially expressed in wild type N2 and *nipi-3(fr4)* (first tab) or *pmk-1(km25)* (second tab) animals fed control OP50 *E. coli* at 20 °C until the L4 stage. Expression values are the average of three independent replicates. Gene ontology terms enriched in each category are listed in the third tab. (XLSX 59 kb)
Additional file 4: Table S3. NanoString analysis of wild type N2 and *nipi-3(fr4)* gene expression. This table lists the gene expression fold changes between wild type N2 and *nipi-3(fr4)* animals fed *P. aeruginosa* PA14 or control OP50 *E. coli* for 6 hours (first tab) or fed *E. coli* expressing ToxA or BL21 control for 24 hours (second tab). Expression values are the average of three (PA14 and OP50) or two (ToxA and BL21 control) independent replicates and normalized to three control genes. Primary data are provided in Additional file 15. (XLSX 33 kb)
Additional file 5: Figure S2. Genes differentially expressed in *nipi-3(fr4)* are enriched for translational inhibitor- and pathogen-response genes. a. Overlaps between genes differentially expressed in *nipi-3(fr4)* versus wild type animals fed the indicated food. Numbers provided for major overlap classes. b. Overlap between genes differentially expressed in *nipi-3(fr4)* versus wild type animals fed control OP50 *E. coli* and genes induced/repressed by ToxA or hygromycin versus control bacteria in wild type animals [5]. *P* < 1 × 10^–95^ (hypergeometric test). c. Overlap between genes differentially expressed in *nipi-3(fr4)* versus wild type animals fed *P. aeruginosa* or ToxA and genes induced/repressed by *P. aeruginosa* or ToxA versus control bacteria in wild type animals *P* < 5 × 10^–6^ (hypergeometric test). Data collected from microarray (b) or NanoString (a, c) analyses. Primary data for panels a and c are provided in Additional file 15. (PDF 403 kb)
Additional file 6: Figure S3. Lifespan and gene expression analysis of *nipi-3(fr4)* and mutants of other immune pathways required for the ToxA response. a. qRT-PCR comparison of wild type N2, *nipi-3(fr4)*, *pmk-1(km25)*, *zip-2(tm4248)*, and *fshr-1(ok778)* animals following exposure to *P. aeruginosa* PA14 or OP50 *E. coli* for 6 hours. Results shown are an average of two (*fshr-1)* or three (remaining samples) biological replicates. b. qRT-PCR comparison of wild type N2, *nipi-3(fr4)*, and *kgb-1(km21)* animals following exposure to *E. coli* expressing ToxA or the BL21 control for 24 hours. Results shown are an average of two biological replicates. For a and b, results are normalized to the value of wild type worms on control *E. coli* for the given gene. Error bars represent SEM. **P* < 0.05 (Student’s t-test) when compared to the corresponding wild type animals. c. Lifespans of wild type N2, *pmk-1(km25)*, *zip-2(tm4248)*, and *nipi-3(RNAi)* fed *E. coli* expressing ToxA. Number of animals scored for each condition was ≥ 55 (257 total). This is a representative experiment of three independent experiments. Primary data for panels a and b are provided in Additional file 15. (PDF 463 kb)
Additional file 7: Table S4. NanoString analysis of wild type N2 gene expression on *P. aeruginosa* or ToxA. This table lists the gene expression fold changes between wild type N2 animals fed *P. aeruginosa* PA14 and animals fed control OP50 *E. coli* for 6 hours or between N2 animals fed *E. coli* expressing ToxA and animals fed the BL21 control for 24 hours. Expression values are the average of three (PA14 and OP50) or two (ToxA and BL21 control) independent replicates and normalized to three control genes. Primary data are provided in Additional file 15. (XLSX 22 kb)
Additional file 8: Table S5. ToxA-induced protein changes. This table lists the proteins differentially expressed in wild type N2 (first tab) or *nipi-3(fr4)* (second tab) animals fed *E. coli* expressing ToxA or the BL21 control for 24 hours. Relative expression levels are an average of two independent replicates. Primary data are provided (third tab). (XLSX 1584 kb)
Additional file 9: Figure S4. Comparing RNA and protein changes in animals exposed to ToxA or control bacteria. Changes of protein and RNA abundances in wild type N2 animals following a 24 hour exposure to *E. coli* expressing ToxA as compared to animals fed a control BL21 *E. coli* (top) or in *nipi-3(fr4*) animals fed control bacteria as compared to similarly treated wild type animals (bottom). Only values with significant protein and/or RNA changes are included. Results shown are an average of two (protein) or three (RNA) biological replicates. Primary data are provided in Additional file 8: Table S5. (PDF 423 kb)
Additional file 10: Table S6. Proteins misregulated in *nipi-3(fr4)* or *pmk-1(km25)* as compared to wild type worms. This table lists the proteins differentially expressed in wild type N2 and *nipi-3(fr4)* animals fed BL21 control bacteria (first tab) or *E. coli* expressing ToxA (second tab) for 24 hours. This table also lists the proteins differentially expressed in wild type N2 and *pmk-1(km25)* animals fed *E. coli* expressing ToxA for 24 hours (third tab). Relative expression levels are an average of two independent replicates. Primary data are provided in Additional file 8. (XLSX 29 kb)
Additional file 11: Table S7. Specificity of tissue-restricted RNAi strains. This table lists the results of RNAi feeding experiments using double stranded RNAs targeting genes expressed in the indicated tissue. Results presented as percentage of affected animals and the total number scored. Synchronized L1 worms were dropped onto the RNAi food and, after 3 days, worms were scored as affected if they developmentally arrested (*act-5*), twitched in 10 mM levamisole (*unc-22*), had a detached cuticle (*bli-1*), low mobility (*unc-45*), sterility (*pos-1*) or any of the previous phenotypes (L4440 control). (XLSX 10 kb)
Additional file 12: Figure S5. Hypodermal immune genes *tpa-1* and *hsp-3* are not hypersusceptible to ToxA. Lifespans of *tpa-1(k530)* and *hsp-3(ok1083)* fed on *E. coli* expressing ToxA or the BL21 control. Number of animals scored for each condition was > 45 (280 total). This is a representative experiment of four independent experiments. (PDF 360 kb)
Additional file 13: Figure S6. Loss of *nipi-3* and *pmk-1* or *kgb-1* results in a shortened lifespan on control food. Lifespans of *pmk-1(km25), pmk-1(km25);nipi-3(RNAi)* (a) or *kgb-1(km21), kgb-1(km21);nipi-3(RNAi)* (b) and *nipi-3(RNAi)* fed on BL21 control *E. coli*. Number of animals scored for each condition was ≥ 160 (482 total; a) and ≥ 140 (381 total; b). These are representative experiments of two independent experiments. (PDF 388 kb)
Additional file 14: Figure S7.
*cebp-1* acts in the intestine and affects immune gene expression. a. Lifespans of MGH167 (gut RNAi) animals grown on equal mixtures of *cebp-1* RNAi and L4440 vector control; *nipi-3* RNAi and L4440 vector control; *cebp-1* and *nipi-3* RNAi; or L4440 vector control alone to the L4 stage. Animals were then transferred to *E. coli* expressing ToxA. Note that the mixed *nipi-3* RNAi showed less ToxA susceptibility than undiluted *nipi-3* RNAi (Fig. S1b). *P* = 0.95 (log-rank test) and 0.4 (Wilcoxon test) for *nipi-3, cebp-1* versus *cebp-1* RNAi; *P* = 0.089 (log-rank test) and 0.0004 (Wilcoxon test) for *nipi-3* versus *cebp-1* RNA. Number of animals scored for each condition was > 65 (426 total). This is a representative experiment of two independent experiments. b. qRT-PCR comparison of the indicated strains exposed to *E. coli* expressing ToxA for 24 hours. Results shown are an average of two biological replicates and are normalized to the corresponding wild type ToxA value. Error bars represent SEM. *nipi-3* refers to *nipi-3(fr4).* Primary data for panel b are provided in Additional file 15. (PDF 206 kb)
Additional file 15: Primary data for Figs. 1b and 3b, Figures S3a, b, and S7b, and Tables S3 and S4. (XLSX 35 kb)

## Abbreviations

FDR: False Discovery Rate
GO: Gene Ontology
ToxA: Exotoxin A
