# Supplementary figures and images for: Tribbles ortholog NIPI-3 and bZIP transcription factor CEBP-1 regulate a Caenorhabditis elegans intestinal immune surveillance pathway
Source: BMC Biol. 2016 Dec 7;14:105. doi: 10.1186/s12915-016-0334-6 (PMC5143455; doi:10.1186/s12915-016-0334-6)

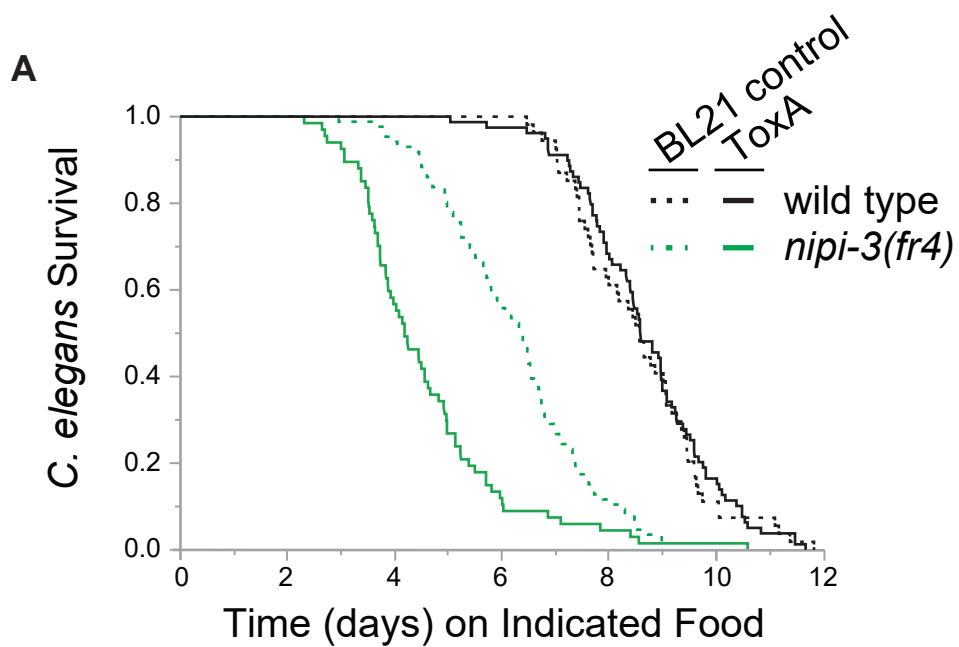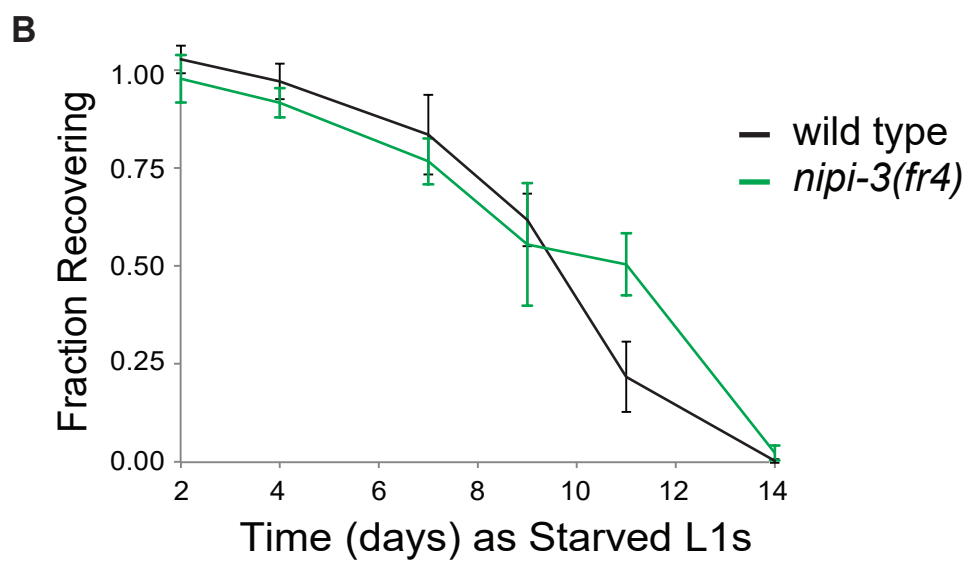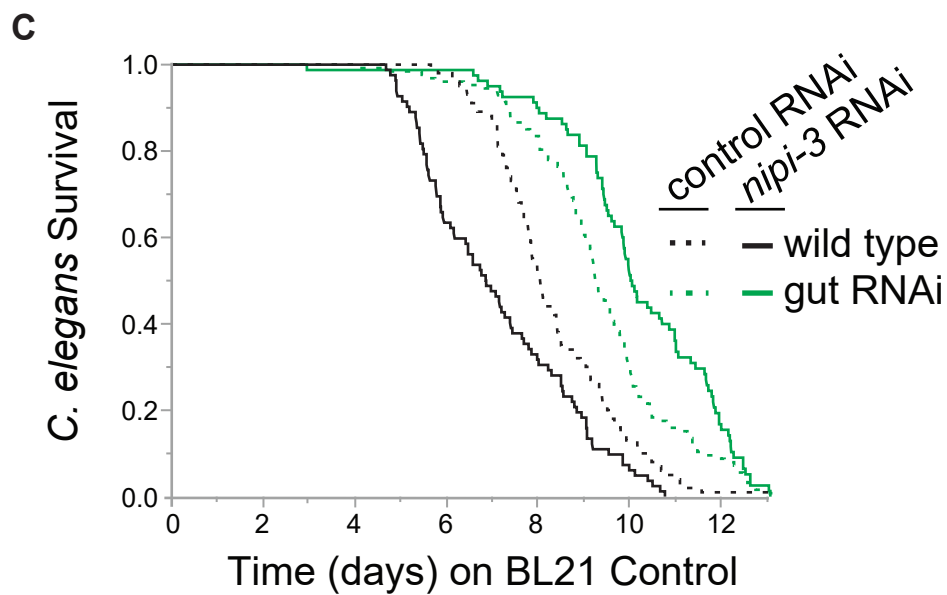

Supplement: Additional file 2: Figure S1. — The ToxA defect of nipi-3(fr4) is not a result of general strain health. a. Lifespans of nipi-3(fr4) and wild type N2 fed E. coli expressing ToxA or the BL21 control. P < 0.001 comparing nipi-3(fr4) ToxA and BL21; P > 0.05 comparing N2 ToxA and BL21 (log-rank test). b. Fraction of nipi-3(fr4) or wild type N2 animals that grow within 2 or 3 days of being fed OP50 following starvation at room temperature for the indicated time. Results shown are an average of six biological replicates. Error bars represent SD. c. Lifespans of wild type N2 and MGH167 (gut RNAi) animals fed on BL21 control E. coli following either nipi-3 or L4440 vector control RNAi. Number of animals scored for each condition was > 65 (286 total; a) and > 65 (332 total; c). These are representative experiments of four (a), three (c), or two (b) independent experiments. (PDF 435 kb) [file 12915_2016_334_MOESM2_ESM.pdf]

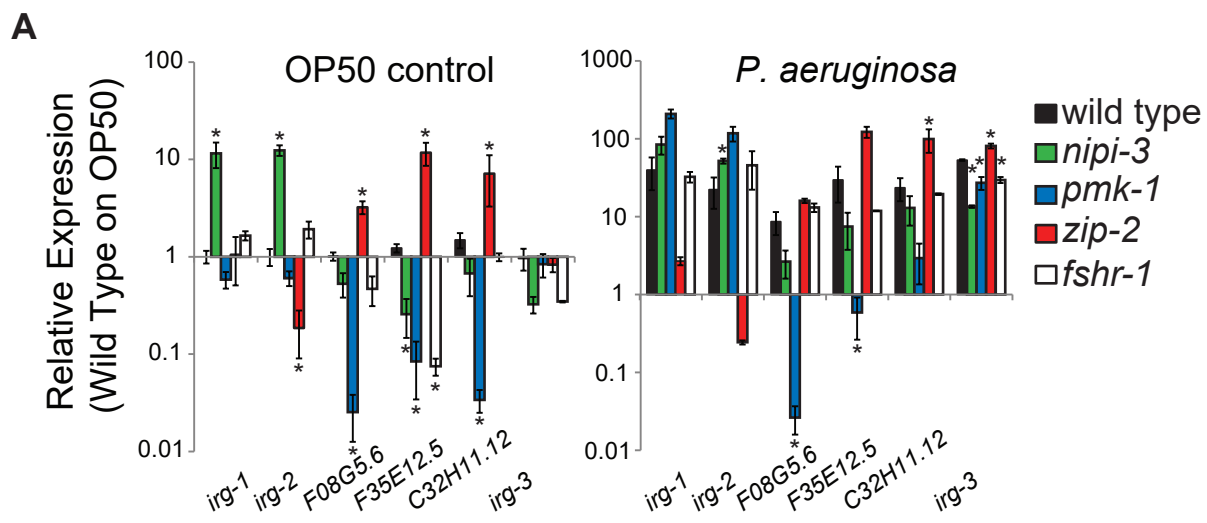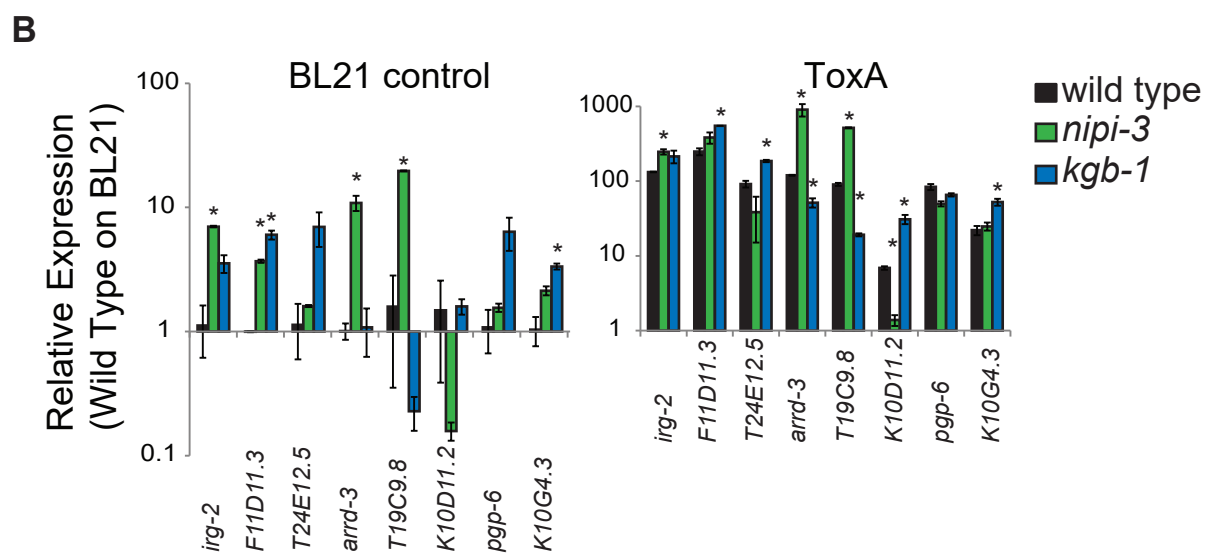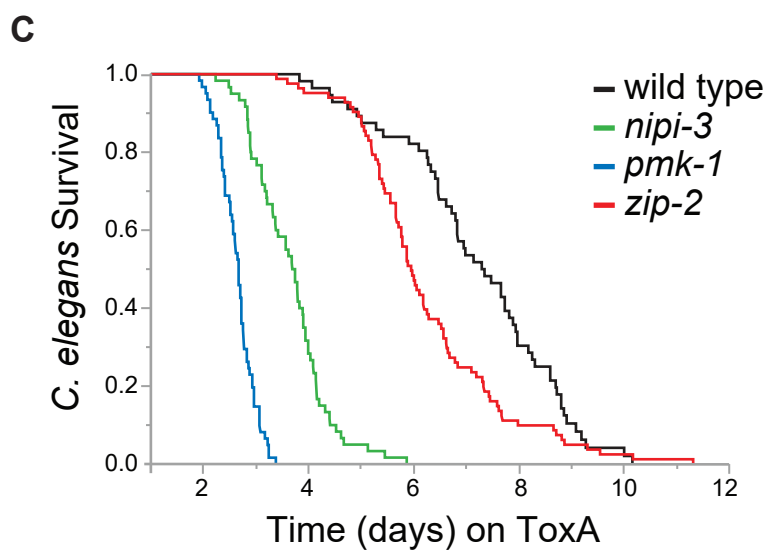

Supplement: Additional file 6: Figure S3. — Lifespan and gene expression analysis of nipi-3(fr4) and mutants of other immune pathways required for the ToxA response. a. qRT-PCR comparison of wild type N2, nipi-3(fr4), pmk-1(km25), zip-2(tm4248), and fshr-1(ok778) animals following exposure to P. aeruginosa PA14 or OP50 E. coli for 6 hours. Results shown are an average of two (fshr-1) or three (remaining samples) biological replicates. b. qRT-PCR comparison of wild type N2, nipi-3(fr4), and kgb-1(km21) animals following exposure to E. coli expressing ToxA or the BL21 control for 24 hours. Results shown are an average of two biological replicates. For a and b, results are normalized to the value of wild type worms on control E. coli for the given gene. Error bars represent SEM. *P < 0.05 (Student’s t-test) when compared to the corresponding wild type animals. c. Lifespans of wild type N2, pmk-1(km25), zip-2(tm4248), and nipi-3(RNAi) fed E. coli expressing ToxA. Number of animals scored for each condition was ≥ 55 (257 total). This is a representative experiment of three independent experiments. Primary data for panels a and b are provided in Additional file 15. (PDF 463 kb) [file 12915_2016_334_MOESM6_ESM.pdf]

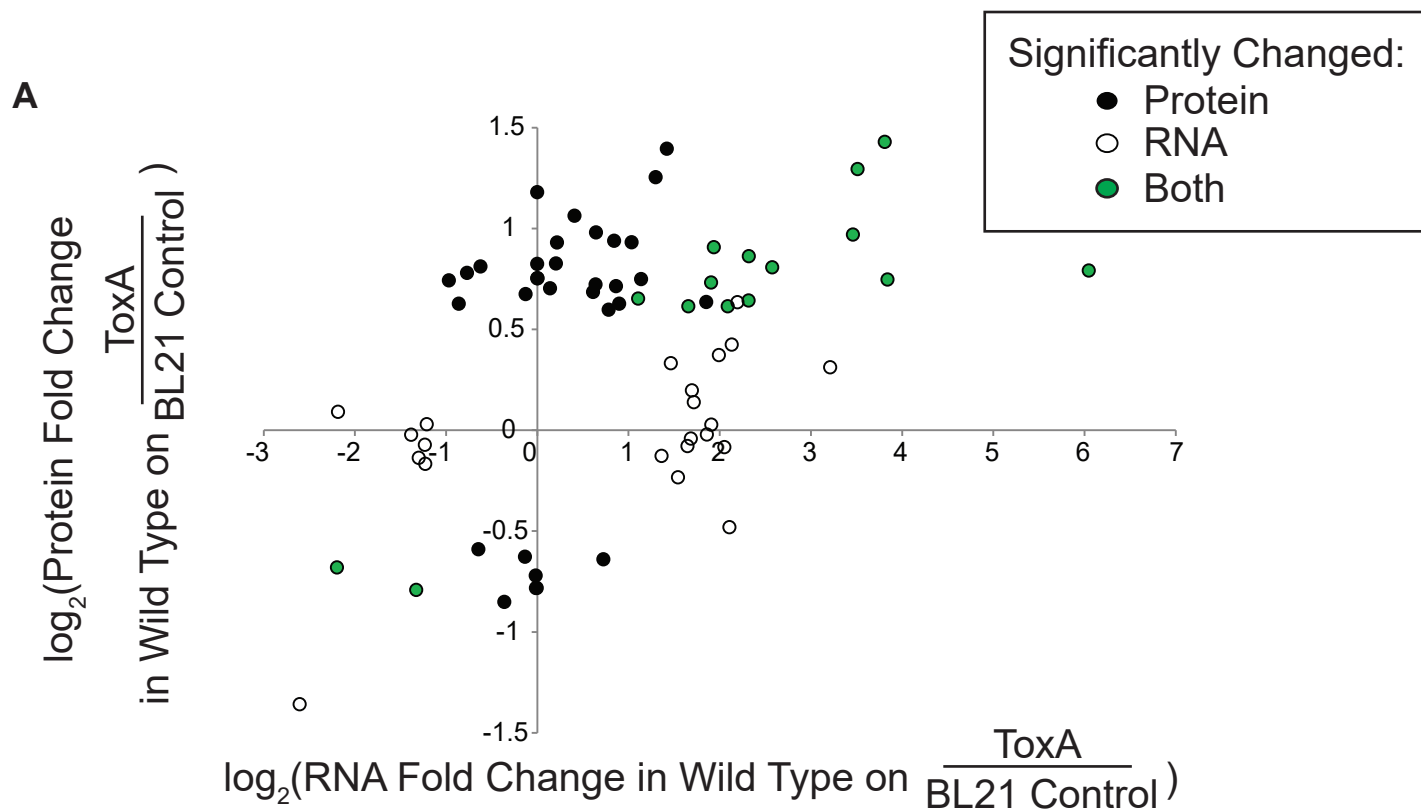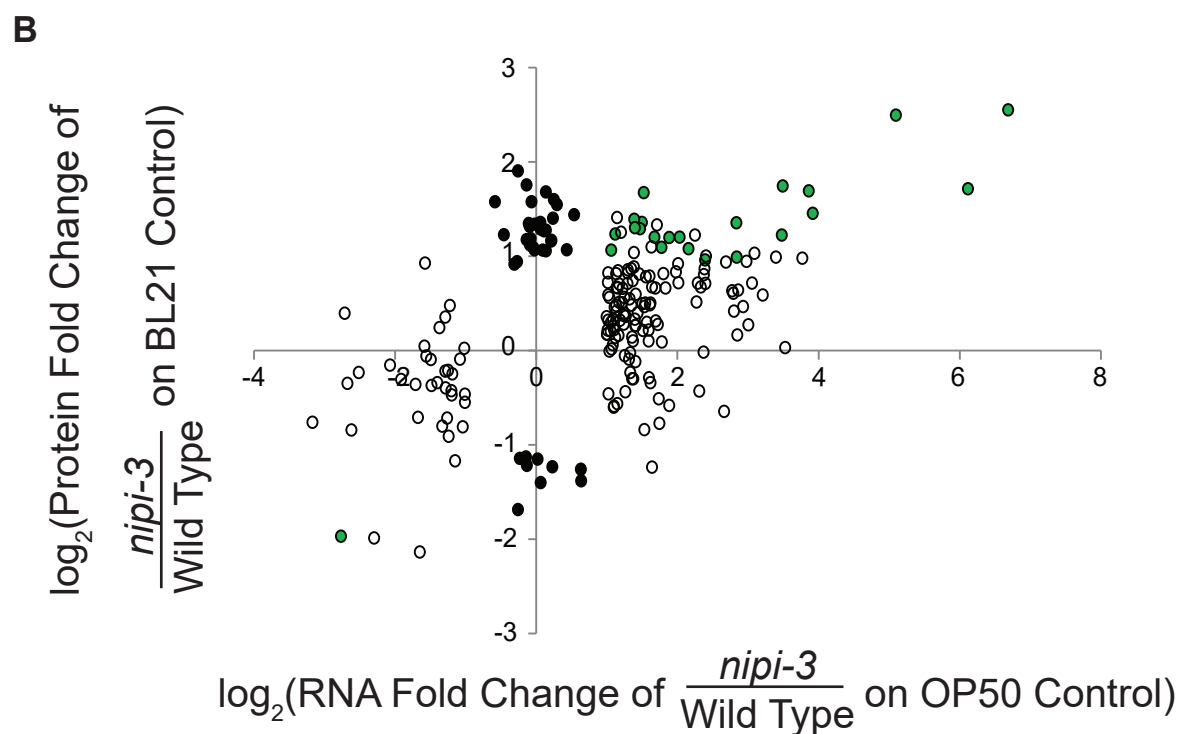

Supplement: Additional file 9: Figure S4. — Comparing RNA and protein changes in animals exposed to ToxA or control bacteria. Changes of protein and RNA abundances in wild type N2 animals following a 24 hour exposure to E. coli expressing ToxA as compared to animals fed a control BL21 E. coli (top) or in nipi-3(fr4) animals fed control bacteria as compared to similarly treated wild type animals (bottom). Only values with significant protein and/or RNA changes are included. Results shown are an average of two (protein) or three (RNA) biological replicates. Primary data are provided in Additional file 8: Table S5. (PDF 423 kb) [file 12915_2016_334_MOESM9_ESM.pdf]

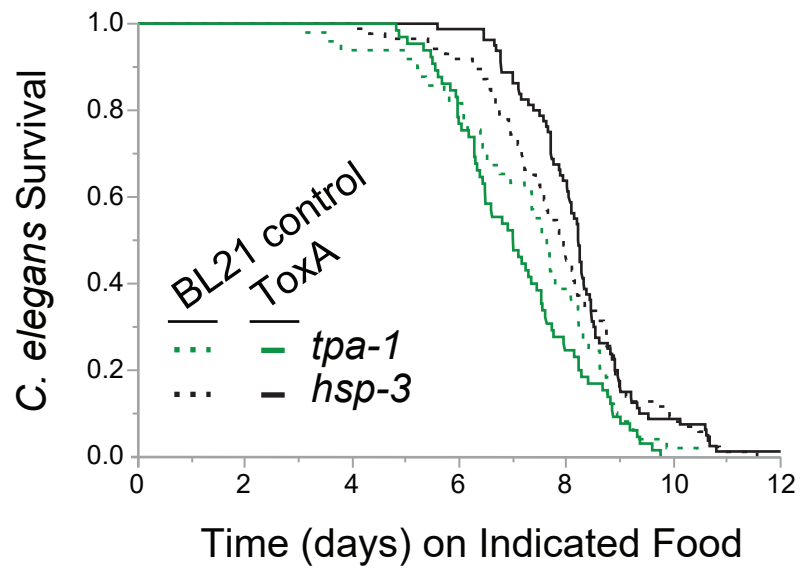

Supplement: Additional file 12: Figure S5. — Hypodermal immune genes tpa-1 and hsp-3 are not hypersusceptible to ToxA. Lifespans of tpa-1(k530) and hsp-3(ok1083) fed on E. coli expressing ToxA or the BL21 control. Number of animals scored for each condition was > 45 (280 total). This is a representative experiment of four independent experiments. (PDF 360 kb) [file 12915_2016_334_MOESM12_ESM.pdf]

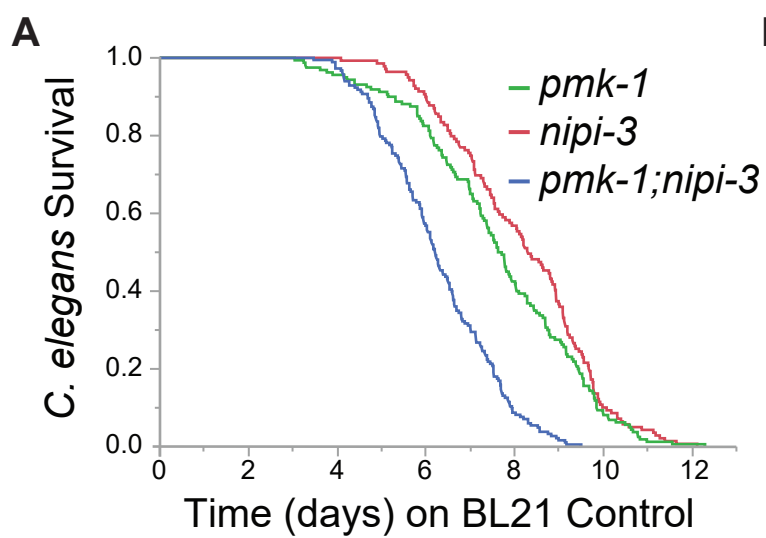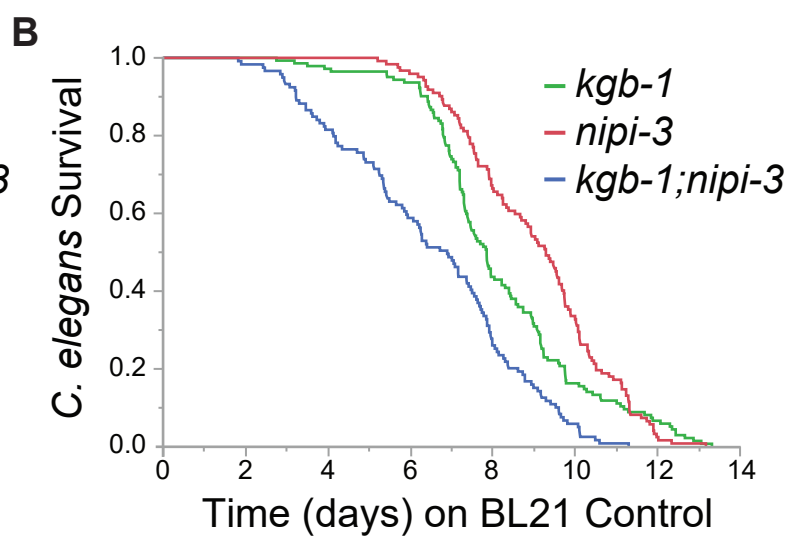

Supplement: Additional file 13: Figure S6. — Loss of nipi-3 and pmk-1 or kgb-1 results in a shortened lifespan on control food. Lifespans of pmk-1(km25), pmk-1(km25);nipi-3(RNAi) (a) or kgb-1(km21), kgb-1(km21);nipi-3(RNAi) (b) and nipi-3(RNAi) fed on BL21 control E. coli. Number of animals scored for each condition was ≥ 160 (482 total; a) and ≥ 140 (381 total; b). These are representative experiments of two independent experiments. (PDF 388 kb) [file 12915_2016_334_MOESM13_ESM.pdf]

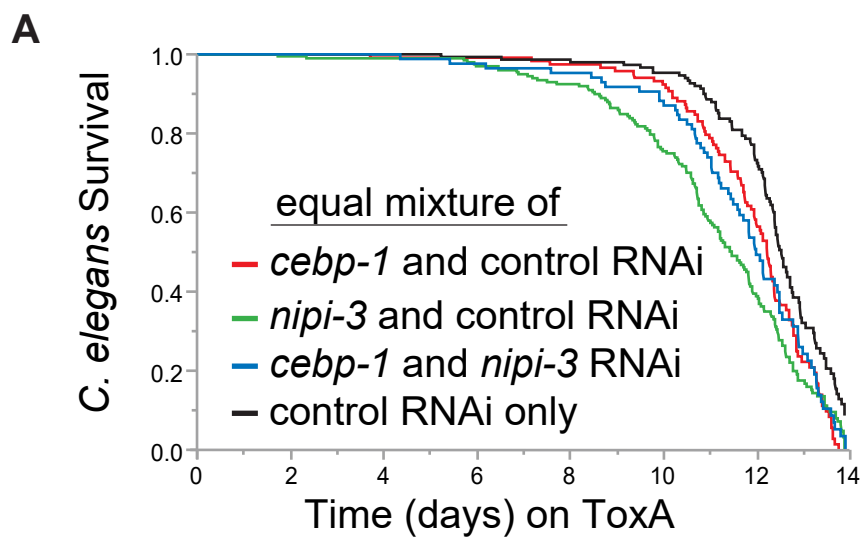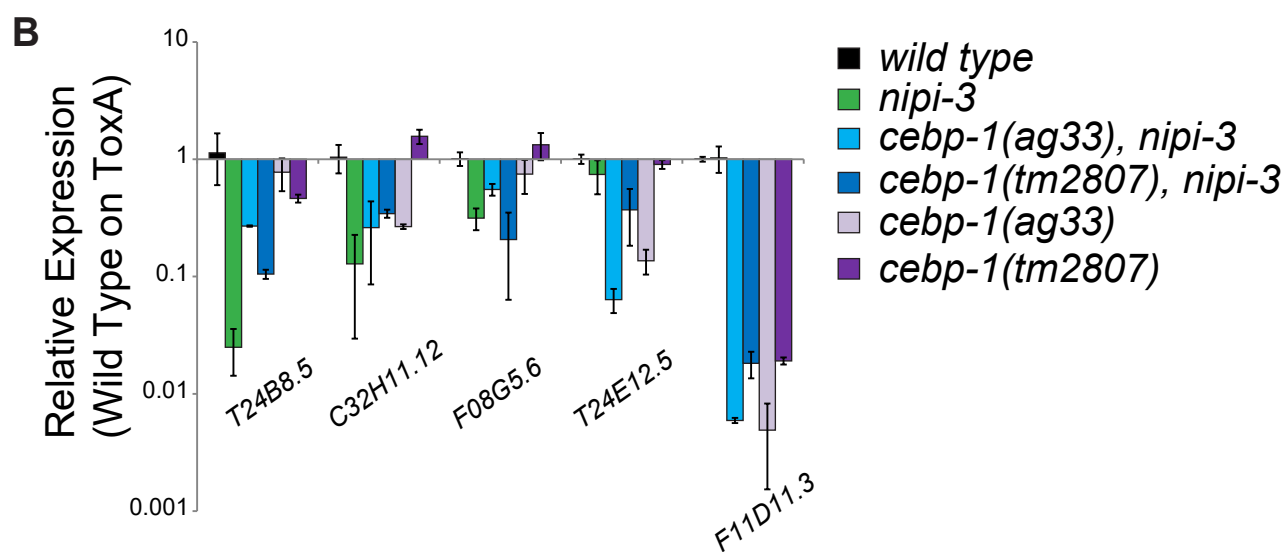

Supplement: Additional file 14: Figure S7. — cebp-1 acts in the intestine and affects immune gene expression. a. Lifespans of MGH167 (gut RNAi) animals grown on equal mixtures of cebp-1 RNAi and L4440 vector control; nipi-3 RNAi and L4440 vector control; cebp-1 and nipi-3 RNAi; or L4440 vector control alone to the L4 stage. Animals were then transferred to E. coli expressing ToxA. Note that the mixed nipi-3 RNAi showed less ToxA susceptibility than undiluted nipi-3 RNAi (Fig. S1b). P = 0.95 (log-rank test) and 0.4 (Wilcoxon test) for nipi-3, cebp-1 versus cebp-1 RNAi; P = 0.089 (log-rank test) and 0.0004 (Wilcoxon test) for nipi-3 versus cebp-1 RNA. Number of animals scored for each condition was > 65 (426 total). This is a representative experiment of two independent experiments. b. qRT-PCR comparison of the indicated strains exposed to E. coli expressing ToxA for 24 hours. Results shown are an average of two biological replicates and are normalized to the corresponding wild type ToxA value. Error bars represent SEM. nipi-3 refers to nipi-3(fr4). Primary data for panel b are provided in Additional file 15. (PDF 206 kb) [file 12915_2016_334_MOESM14_ESM.pdf]
