# Supplementary material for: Tribbles ortholog NIPI-3 and bZIP transcription factor CEBP-1 regulate a Caenorhabditis elegans intestinal immune surveillance pathway
Source: BMC Biol. 2016 Dec 7;14:105. doi: 10.1186/s12915-016-0334-6 (PMC5143455; doi:10.1186/s12915-016-0334-6)
Supplement: Additional file 5: Figure S2. — Genes differentially expressed in nipi-3(fr4) are enriched for translational inhibitor- and pathogen-response genes. a. Overlaps between genes differentially expressed in nipi-3(fr4) versus wild type animals fed the indicated food. Numbers provided for major overlap classes. b. Overlap between genes differentially expressed in nipi-3(fr4) versus wild type animals fed control OP50 E. coli and genes induced/repressed by ToxA or hygromycin versus control bacteria in wild type animals [5]. P < 1 × 10^–95^ (hypergeometric test). c. Overlap between genes differentially expressed in nipi-3(fr4) versus wild type animals fed P. aeruginosa or ToxA and genes induced/repressed by P. aeruginosa or ToxA versus control bacteria in wild type animals P < 5 × 10^–6^ (hypergeometric test). Data collected from microarray (b) or NanoString (a, c) analyses. Primary data for panels a and c are provided in Additional file 15. (PDF 403 kb) [file 12915_2016_334_MOESM5_ESM.pdf]

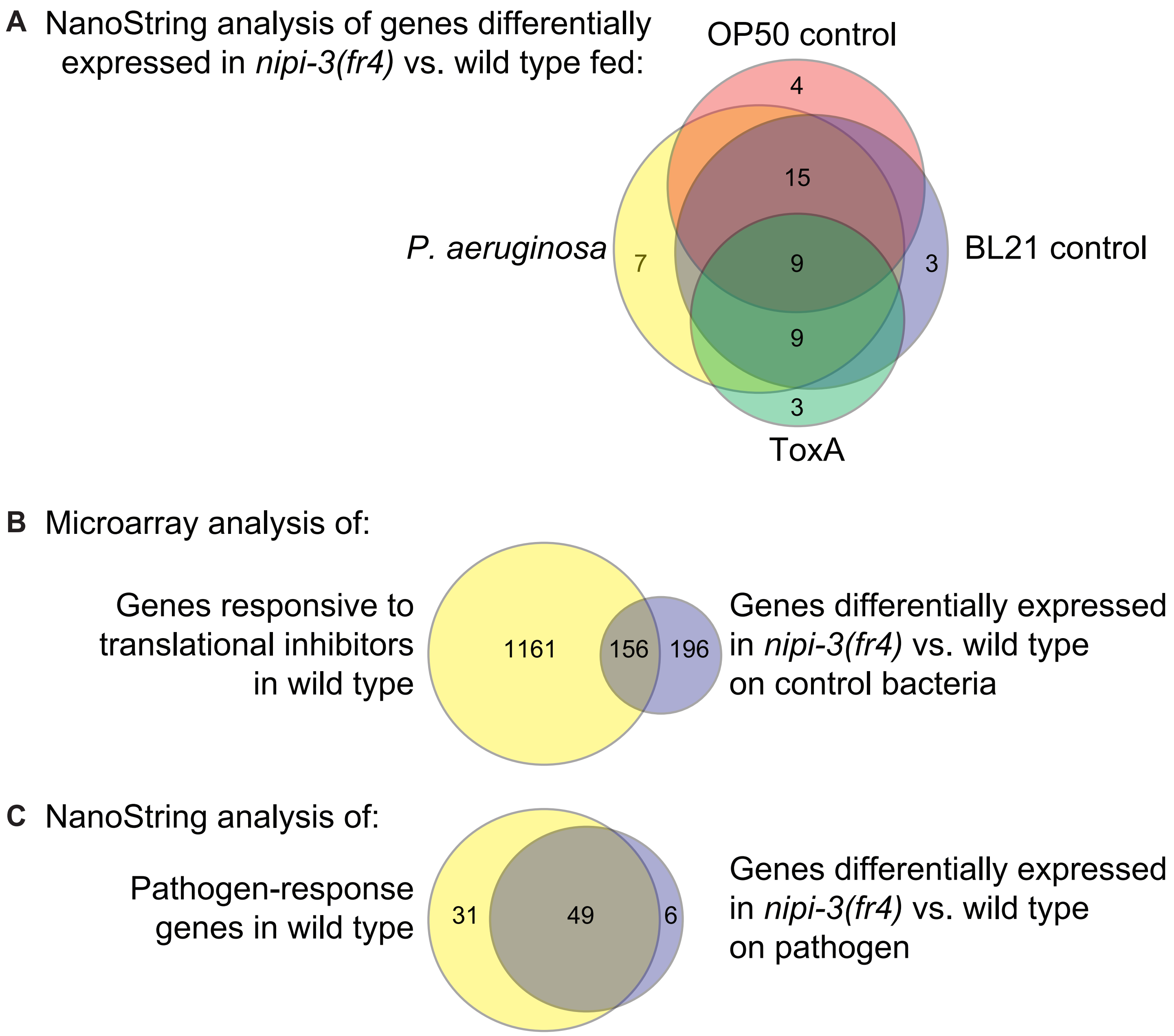
A NanoString analysis of genes differentially expressed in *nipi-3(fr4)* vs. wild type fed:
OP50 control
4
15
P. aeruginosa
7
9
3
BL21 control
9
3
ToxA
B Microarray analysis of:
Genes responsive to translational inhibitors in wild type
1161
156
196
Genes differentially expressed in *nipi-3(fr4)* vs. wild type on control bacteria
C NanoString analysis of:
Pathogen-response genes in wild type
31
49
6
Genes differentially expressed in *nipi-3(fr4)* vs. wild type on pathogen
